# Structural Basis of Stereospecificity in the Bacterial Enzymatic Cleavage of β-Aryl Ether Bonds in Lignin*[Fn FN2]

**DOI:** 10.1074/jbc.M115.694307

**Published:** 2015-12-04

**Authors:** Kate E. Helmich, Jose Henrique Pereira, Daniel L. Gall, Richard A. Heins, Ryan P. McAndrew, Craig Bingman, Kai Deng, Keefe C. Holland, Daniel R. Noguera, Blake A. Simmons, Kenneth L. Sale, John Ralph, Timothy J. Donohue, Paul D. Adams, George N. Phillips

**Affiliations:** From the ‡ Department of Biochemistry, University of Wisconsin, Madison, Wisconsin 53706,; the § United States Department of Energy Great Lakes Bioenergy Research Center, Wisconsin Energy Institute, University of Wisconsin, Madison, Wisconsin 53726,; the ¶ Joint BioEnergy Institute, Emeryville, California 94608,; the ‖ Physical Biosciences Division, Lawrence Berkeley National Laboratory, Berkeley, California 94720,; the Departments of ** Civil and Environmental Engineering and; §§ Bacteriology, University of Wisconsin, Madison, Wisconsin 53706,; the ‡‡ Biological and Engineering Sciences Center, Sandia National Laboratories, Livermore, California 94551,; the ¶¶ Department of Bioengineering, University of California, Berkeley, California 94720, and; the ‖‖ Department of Biochemistry and Cell Biology, Rice University, Houston, Texas 77251

**Keywords:** enzyme catalysis, enzyme mechanism, enzyme structure, lignin degradation, plant cell wall, protein structure, stereoselectivity, X-ray crystallography, structural enzymology

## Abstract

Lignin is a combinatorial polymer comprising monoaromatic units that are linked via covalent bonds. Although lignin is a potential source of valuable aromatic chemicals, its recalcitrance to chemical or biological digestion presents major obstacles to both the production of second-generation biofuels and the generation of valuable coproducts from lignin's monoaromatic units. Degradation of lignin has been relatively well characterized in fungi, but it is less well understood in bacteria. A catabolic pathway for the enzymatic breakdown of aromatic oligomers linked via β-aryl ether bonds typically found in lignin has been reported in the bacterium *Sphingobium* sp. SYK-6. Here, we present x-ray crystal structures and biochemical characterization of the glutathione-dependent β-etherases, LigE and LigF, from this pathway. The crystal structures show that both enzymes belong to the canonical two-domain fold and glutathione binding site architecture of the glutathione *S*-transferase family. Mutagenesis of the conserved active site serine in both LigE and LigF shows that, whereas the enzymatic activity is reduced, this amino acid side chain is not absolutely essential for catalysis. The results include descriptions of cofactor binding sites, substrate binding sites, and catalytic mechanisms. Because β-aryl ether bonds account for 50–70% of all interunit linkages in lignin, understanding the mechanism of enzymatic β-aryl ether cleavage has significant potential for informing ongoing studies on the valorization of lignin.

## Introduction

The primary obstacle in the production of lignocellulosic biofuels is the release of sugars in high quantities at low cost from recalcitrant biomass feedstocks (1). Lignin is the prime source of this recalcitrance, and there has been renewed interest in the microbial enzymes capable of lignin degradation and catabolism of lignin-derived compounds (2, 3). Generally, white rot fungi secrete lignin peroxidases, versatile peroxidase, manganese peroxidases, and laccases that are involved in the initial degradation of lignin (4, 5), whereas bacteria are thought to play a role in further degradation of lignin-derived lower molecular weight compounds (6).

**FIGURE 1.**
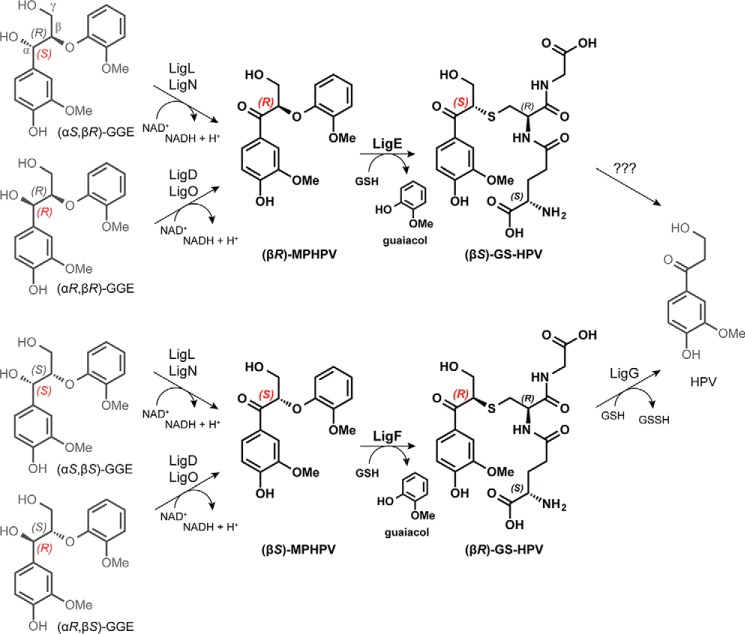
**The *Sphingobium* sp. strain SYK-6 β-etherase pathway.** Chiral carbons at which stereospecific reactions occur are highlighted (*red*). Stereospecific reactions for (α*S*,β*R*)-GGE and (α*S*,β*S*)-GGE oxidation (by LigL and LigN) and for (α*R*,β*R*)-GGE and (α*R*,β*S*)-GGE oxidation (by LigD and LigO), the GSH-dependent stereospecific cleavage reactions of (β*R*)-MPHPV (by LigE) and (β*S*)-MPHPV (by LigF), and the stereospecific lyase reaction of LigG with (β*S*)-GS-HPV are shown.

*Sphingobium* sp. strain SYK-6, one of the most well studied bacteria implicated in lignin-derived compound degradation, has the ability to grow on a wide variety of dimeric aromatic compounds representing the various units, with their characteristic interunit linkages, present in plant lignins (6, 7). The cleavage of β-aryl ether (termed simply β-ether hereafter) linkages is an essential step in any catabolic process for degradation of lignin-derived aromatic oligomers, because this bond type accounts for 50–70% of all interunit linkages in lignin polymers (8). Using a β-ether-linked phenolic lignin model substrate, guaiacylglycerol-β-guaiacyl ether (GGE^5^; Fig. 1), three enzymatic reactions composing the β-ether degradation pathway were identified in *Sphingobium* sp. strain SYK-6 (7, 9, 10). Following oxidation of the α-hydroxyl group in GGE by a Cα-dehydrogenase, stereospecific glutathione (GSH)-dependent cleavage of the β-ether linkage in β-(3′-methoxyphenoxy)-γ-hydroxypropiovanillone (MPHPV) is catalyzed by the glutathione *S*-transferase (GST) enzymes LigE and LigF, forming β-glutathionyl-γ-hydroxypropiovanillone (GS-HPV) and guaiacol. LigE catalyzes stereospecific cleavage of (β*R*)-MPHPV to (β*S*)-GS-HPV, whereas LigF catalyzes the cleavage of (β*S*)-MPHPV to (β*R*)-GS-HPV. Finally, GSH-dependent and stereospecific elimination of GSH from (β*S*)-GS-HPV is catalyzed by the GST lyase LigG, generating glutathione disulfide (GSSG) and the achiral derivative γ-hydroxypropiovanillone, which ultimately serves as the growth substrate for strain SYK-6 (6, 10) (Fig. 1). The fate of the corresponding (*R*)-stereoisomer of GS-HPV is not presently understood. Recently, it has been reported that these GST family member enzymes have the ability to work with lignin-derived materials *in vitro* (11, 12).

GST superfamily members are multifunctional enzymes often involved in cellular detoxification processes via GSH conjugation (13). However, some bacterial GSTs are implicated in basal metabolism and supply bacterial cells with carbon (14). GSTs with >40% sequence identity are traditionally considered to be in the same class, whereas proteins of different classes have typically <25% protein sequence identity (15). However, these classifications are also based on a number of other considerations, including structure, function, and biochemical properties (15). Although there are seven classes of GSTs in mammals (Alpha, Mu, Pi, Sigma, Theta, Omega, and Zeta), there is an ever-increasing number of non-mammalian classes, including Beta, Chi, Delta, Epsilon, Lambda, Phi, and Tau, as well as a number of more recently defined novel classes (15–17). Previous studies have suggested that the β-etherase enzymes LigE and LigF might be classified in the fungal GSTFuA class of GSTs based on sequence phylogeny (18).

Because plant lignins are racemic polymers, complementary stereospecificities of the multiple enzymes in the β-ether degradation pathway are required by bacteria to oxidize and cleave the various stereoisomers that are present in lignin polymers (19–22). Here, we describe three protein crystal structures and provide the corresponding biochemical data for the LigE and LigF enzymes involved in the β-ether cleavage step of the *Sphingobium* sp. strain SYK-6 degradation pathway. The modest structural homology of these two enzymes highlights the fitness adaptation afforded in this and probably other microbial catabolic pathways that can degrade lignin-derived materials, required for enzymatic degradation of such racemic products. This work provides new insights into the structure-function relationships and biochemistry of this pathway, expanding our knowledge of the bacterial catabolism of lignin-derived compounds. Because lignin is the most abundant aromatic polymer in nature, this study informs broader lignin valorization efforts that will ultimately enable the development of efficient pathways for the conversion of lignin into renewable aromatics with applications in advanced biofuels and chemicals (23).

## Experimental Procedures

### 

#### 

##### Gene Cloning

LigE was synthesized and cloned into a custom vector (pCPD) assembled by GenScript (Piscataway, NJ). This vector combined the pVP16 backbone (provided by the Center for Eukaryotic Structural Genomics, Madison, WI) with the gene of interest and a C-terminal fusion protein tag containing the *Vibro cholera* MARTX toxin cysteine protease domain (CPD) (24). During protein purification, the CPD tag can be activated by the addition of inositol hexakisphosphate, cleaving at a leucine positioned between the N terminus protein of interest and CPD. The pVP80K_LigFΔ242 vector was prepared using polymerase incomplete primer extension as described previously using Phusion High-Fidelity PCR master mix with HF buffer (New England Biolabs Inc., Ipswich, MA), and primers from Integrated DNA Technologies (Coralville, IA) (25). The pVP80K vector was provided by the Center for Eukaryotic Structural Genomics (Madison, WI), and the pVP102KSSLigF vector containing full-length wild type LigF was prepared as described previously (9). Insert and vector backbone PCR products were mixed 1:1 and immediately transformed into *Escherichia coli* One Shot® TOP10 cells (Invitrogen). The pVP80K_LigFΔ242 vector was purified from *E. coli* (One Shot® TOP10, 10 ml of LB with kanamycin, 18 h at 37 °C) using the QIAprep® spin miniprep kit (Qiagen, Germantown, MD) and transformed into the laboratory strain *E. coli* B834(DE3) Z-competent cells (Zymo Research, Orange, CA).

##### Enzyme Expression and Purification

NEB Express protein expression cells (New England Biolabs Inc., Ipswich, MA) containing pCPD-LigE were grown in autoinducing selenomethionine medium as described previously (26) and harvested via centrifugation. Harvested cells were resuspended in 30 ml of lysis buffer (50 mm HEPES buffer, pH 7.4, 150 mm NaCl, and 40 mm imidazole) and lysed by an Avestin EmulsiFlex-C3 homogenizer. The C-terminally His-tagged proteins were purified from the clarified supernatant using precharged nickel-IMAC resin (GE Healthcare). After protein binding and washing twice with lysis buffer, inositol hexakisphosphate was added to a final concentration of 200 μm. Note that the inositol hexakisphosphate was first diluted to 10 mm in lysis buffer to neutralize the acidic pH of the stock solution. After 1 h of incubation, the resin was washed with 1 ml of lysis buffer to elute the cleaved protein. Following buffer exchange into 20 mm Tris, pH 8, the LigE protein was further purified using a HiTrap Q HP anion exchange column. Fractions containing LigE, as confirmed by SDS-PAGE, were pooled and concentrated. Final protein cleanup was done using gel filtration on a Superdex 200 10/300 GL column (GE Healthcare).

Laboratory strain *E. coli* B834(DE3) Z-competent cells (Zymo Research, Orange, CA) containing the pVP80K_LigFΔ242 plasmid were grown in autoinducing selenomethionine medium as described previously (26) and harvested via centrifugation. Harvested cells were resuspended in 20 ml of lysis buffer (20 mm sodium phosphate buffer, pH 7.5, 500 mm sodium chloride, 20% ethylene glycol) and lysed by sonication. The N-terminally His-tagged LigFΔ242 fusion protein was purified from the supernatant by immobilized nickel affinity chromatography using a HiTrap Q HP anion exchange column on an ÄKTA FPLC system (GE Healthcare, Piscataway, NJ). Fractions containing LigFΔ242, as determined by SDS-PAGE, were combined and dialyzed overnight at 4 °C. LigFΔ242 was cleaved from the fusion protein using tobacco etch virus protease (1 mg/100 mg of protein; provided by the Center for Eukaryotic Structural Genomics). Following cleavage, LigFΔ242 and the polyhistidine tag were separated using a HiTrap Q HP anion exchange column. Pooled fractions containing LigFΔ242, as confirmed by SDS-PAGE, were pooled and concentrated to 3 ml. Final size exclusion purification was performed on a HiLoad^TM^ 26/60 Supradex^TM^ 200 preparation grade column.

##### Enzyme Kinetic Assays

*In vitro* β-etherase assays with LigE, LigF, LigFΔ242, and LigFΔ242-S13A were conducted in an aqueous assay buffer (25 mm Tris, 2.5% DMSO, 5 mm GSH, pH 7.0–10.0) at 30 °C with an initial substrate concentration of 1.5 mm and enzyme concentrations of either 160 nm (LigE), 170 nm (LigF), 180 nm (LigFΔ242), 3.9 μm (LigFΔ242-S13A), 11.2 μm (LigE with (β*S*)-F-FPHPV), or 12.0 μm (LigF with (β*R*)-F-FPHPV). Enantiopure preparations of (β*R*)-FPHPV and (β*S*)-FPHPV were obtained from chiral chromatographic separation of the parent racemate as described previously (9). Similarly, chiral chromatography was used for the separation of (β*S*)-F-FPHPV and (β*R*)-F-FPHPV, with (β*S*)-F-FPHPV being used as a substrate in the LigE assays. Synthesis and purification details for enzymatic substrates FPHPV and F-FPHPV are described in the supplemental material.

Michaelis-Menten curves were generated by measuring the enzymatic specific activities over a range of initial substrate concentrations (1.50, 1.25, 1.00, 0.75, 0.50, and 0.25 mm) obtained from serial dilution of a 1.5 mm substrate buffer made immediately prior to conducting the assays. The 1-ml assays were conducted in triplicate and were managed as follows: 1) the substrate was dissolved in DMSO at 60 mm, and 25 μl were added to a 2-ml vial; 2) 875 μl of 25.7 mm Tris, pH *X*, was added (where *X* is higher than the intended pH of the assay to account for the acidic effect of GSH (*e.g.* pH *X* = 11.5 drops to pH 8.0 after the addition of 5 mm GSH); 3) 50 μl of 100 mm GSH was added (100 mm GSH stock solution was prepared by adding GSH to 25 mm Tris (pH *X*)); 4) 50 μl of 20× concentrated enzyme was added; 5) 150-μl samples were collected after 0, 6, 12, 18, 24, and 30 s of incubation, and enzymatic activity was abolished by pipetting each sample into 5 μl of 5 m phosphoric acid; and 6) the remaining reaction volume was used to measure the pH of the mixture with pH paper.

Each sample was then subjected to C_18_-reversed phase HPLC using a Beckman 125NM solvent delivery module equipped with a Beckman 168 UV detector. Samples and external standards were quantified by UV absorption at 280 nm. The HPLC mobile phase was a mixture of aqueous buffer (5 mm formic acid in 95:5 water/acetonitrile) and methanol at a flow rate of 1.0 ml/min. The ratio of buffers was adjusted as follows: 0–6 min, 30% methanol; 6–15 min, gradient from 30 to 80% methanol; 15–25 min, 80% methanol; 25–26 min, gradient from 80 to 30% methanol; 26–33 min, 30% methanol. Vanillin concentrations were quantified for each time point, and a linear regression was generated over the 30-s assay period in order to calculate the specific activity of each reaction. Averages of the triplicate assays were reported.

##### Crystallization

LigE was concentrated to 9 mg ml^−1^ and dialyzed against 20 mm HEPES, pH 7.4, and 50 mm NaCl. LigF was dialyzed in 10 mm HEPES buffer, pH 7.5, containing 50 mm sodium chloride, 0.5 mm tris(2-carboxyethyl)phosphine, and 1 mm GSH, and concentrated to 18.5 mg ml^−1^. LigE and LigF proteins were screened using the sparse matrix method (27) with a Phoenix Robot (Art Robbins Instruments, Sunnyvale, CA) and a Mosquito dispenser (TTP LabTech, Melbourn, UK) utilizing the following crystallization screens: Berkeley Screen (Lawrence Berkeley National Laboratory), Crystal Screen, SaltRx, PEG/Ion, Index and PEGRx (Hampton Research, Aliso Viejo, CA), and JSCG-plus HT-96 and PACT premier HT-96 (Molecular Dimensions, Altamonte Springs, FL). The optimum conditions for crystallization of the different pathway proteins were found as follows: LigE, 0.1 m ammonium citrate, 0.1 m MES, pH 5.5, 20% PEG 3,350, and 5% isopropyl alcohol; LigF, 25% polyethylene glycol monomethyl ether 2000, 0.25 m trimethylamine *N*-oxide, and 0.1 m Tris, pH 8.5. LigE crystals were obtained after 2–7 days by the sitting drop vapor diffusion method with the drops consisting of a mixture of 0.2 μl of protein solution and 0.2 μl of reservoir solution. LigF crystals were obtained in <24 h with drops containing a mixture of 1 μl of protein solution, 0.8 μl of reservoir solution, and 0.2 μl of seed crystals (pulverized LigFΔ242 crystals in 0.2 m magnesium formate, 30% polyethylene glycol 3350, and 1 mm GSH).

##### X-ray Data Collection and Structure Determination

The LigE crystals were placed in a reservoir solution containing 10–20% (v/v) glycerol and then flash-cooled in liquid nitrogen. The x-ray data sets for LigE were collected at the Berkeley Center for Structural Biology beamlines 8.2.1 and 8.2.2 of the Advanced Light Source at Lawrence Berkeley National Laboratory. LigF crystals were cryoprotected with a reservoir solution containing 30% polyethylene glycol monomethyl ether 2000 and 1 mm GSH. X-ray diffraction data were collected at Life Sciences Collaborative Access Team Sector 21 with x-ray wavelength 0.9793 at the Advanced Photon Source at Argonne National Laboratory. Data sets were indexed and scaled using HKL2000 (28). The LigF crystal structure was determined by molecular replacement using the program *PHASER* (29) within the *Phenix* suite (30) with the coordinates of a LigF homologue (Lig37), whose sequence was identified from a metagenomic analysis of a rice-straw-enriched compost microbial community (Berkeley, CA) (31, 32). The crystal structure of LigE was solved using selenomethionine-labeled protein by the single-wavelength anomalous dispersion method (33) with the *phenix.autosol* (34) and *phenix.autobuild* (35) programs. Structure refinement was performed using the *phenix.refine* program (36). Manual rebuilding using *COOT* (37) and the addition of water molecules allowed construction of the final models. Root mean square deviation differences from ideal geometries for bond lengths, angles, and dihedrals were calculated with *Phenix* (30). The overall stereochemical quality of all final models was assessed using the program *MOLPROBITY* (38), and all figures were generated in PyMOL (39). Structures were observed and analyzed using a stereoscopic television display (40).

##### Small Angle X-ray Scattering

LigE and LigF were dialyzed for 15 h at 4 °C into buffer containing 10 mm HEPES, pH 7.5, 50 mm sodium chloride, 1 mm GSH, and 0.5 mm tris(2-carboxyethyl)phosphine. Prior to data collection, samples were filtered through a 0.2-μm syringe filter and diluted to the working concentrations. After dilution, samples were clarified via centrifugation. The buffer blank was also syringe-filtered and clarified by centrifugation. Small angle scattering data were collected on a Bruker NANOSTAR x-ray generator located at the National Magnetic Resonance Facility at the University of Wisconsin (Madison, WI). Three data collections of 1 h each were taken for each sample and buffer. Data were merged and indexed using the Bruker NANOSTAR small angle x-ray scattering system software (Bruker AXS, Madison, WI). The scattering intensity was obtained by subtracting the scattering of the buffer blank from the sample scattering using the PRIMUS software (41). All SAXS data were processed using GNOM, integrated in the PRIMUS software, to obtain the pair distance distribution function (42). The GNOM output was used with DAMMIF to calculate 10 *ab initio* dummy atom models (43). Models were averaged using DAMAVER and aligned to x-ray crystal structures using SUPCOMB (44, 45). Theoretical scattering curves for the x-ray crystal structure of LigE and a model of the dimer of LigF were calculated using CRYSOL (46).

##### Molecular Docking

Docking of MPHPV to the LigFΔ242-GSH structure was performed using the SwissDock server (47, 48). Docking was performed using the “Accurate” parameter and otherwise default parameters, with the search space limited to a 10 × 10 × 10Å region around the GSH binding. Both the protein and the MPHPV ligand were rigid during docking. The structure of MPHPV was built in ChemDraw (49), converted to three-dimensional coordinates using OpenBable (50). Docking results were visualized and screened using the UCSF Chimera molecular modeling system (51).

## Results

### 

#### 

##### Structural Analysis

Attempts to solve the structure of full-length wild-type LigE (282 residues) and LigF (254 residues) were unsuccessful, but C-terminal truncation constructs of both proteins were generated, successfully crystallized, and used for structural analysis. Truncations of LigE and LigF were designed based on homology models generated by I-TASSER Online and disorder predictions generated using PONDR (52, 53). LigEΔ255 and the LigEΔ255-GSH complex crystallized in the space group C2 with four molecules in the asymmetric unit with electron density for the bound GSH molecule. LigFΔ242-GSH crystallized in the space group P6_3_22 with one molecule in the asymmetric unit. Well defined electron density corresponding to the GSH molecule is also visible in the structure. Data collection, refinement, and model statistics for LigE and LigF are summarized in Table 1.

**TABLE 1:** **LigE and LigF statistics** Summary of crystal parameters, data collection, and refinement statistics. Values in parentheses are for the highest resolution shell.

	LigFΔ242-GSH	LigEΔ255	LigEΔ255-GSH
**Crystal parameters**			
Space group	P6322	C2	C2
Unit cell parameters			
*a*, *b*, *c* (Å)	123.71, 123.71, 66.42	122.55, 97.15, 131.38	121.00, 96.13, 126.16
β		106.65	81.52

**Data collection statistics**			
Wavelength (Å)	0.97857	0.999	1.000
Resolution range (Å)	50.00–2.07 (2.11–2.07)	50–1.90 (1.93–1.90)	50–2.6 (2.65–2.60)
No. of reflections (measured/unique)	326,246/18,884	575,459/114,153	160,219/42,163
Completeness (%)	99.8 (98.3)	99.2 (99.9)	97.7 (93.8)
*R*_merge_*^a^*	0.076 (0.637)	0.137 (0.63)	0.135 (0.61)
Redundancy	17.3 (12.9)	5.0 (4.9)	3.8 (3.6)
Mean *I*/σ(*I*)	9.1 (3.8)	9.0 (1.6)	6.1 (2.5)

**Refinement and model statistics**			
Resolution range (Å)	40.49–2.07 (2.17–2.07)	48–1.90 (1.95–1.90)	48–2.60 (2.65–2.60)
No. of reflections (work/test)	17,278/964	114,138/1,999	42,158/2,000
*R*_cryst_*^b^*	0.161 (0.182)	0.227 (0.289)	0.222 (0.266)
*R*_free_*^c^*	0.214 (0.263)	0.271 (0.350)	0.267 (0.290)
Root mean square deviation bonds (Å)	0.008	0.004	0.003
Root mean square deviation angles (degrees)	1.022	0.956	0.776
*B* factor (protein/solvent) (Å^2^)	39.58/43.93	29.13/37.54	34.22/27.38
*B* factor (GSH) (Å^2^)	26		146, 148, 149, 146
No. of protein atoms	1,983	9,405	8,491
No. of waters	229	1,159	165
Auxiliary molecules (real space correlation coefficient (*CC*))	1 glutathione (0.97), 1 Tris (0.95), 1 PEG (0.95)		4 glutathione (1 per chain, *A* = 0.71, *B* = 0.58, *C* = 0.58, *D* = 0.61)

**Ramachandran plot**			
Favorable region	98.4	95.8	94.4
Additional allowed region	1.6	3.0	4.3
Disallowed region	0	1.2	1.3
Protein Data Bank entry	4XT0	4YAM	4YAN

*^a^ R*_merge_ = Σ*_h_*Σ*_i_*|*I_i_*(*h*) − 〈*I*(*h*)〉|/Σ*_h_*Σ*_i_ I_i_*(*h*), where *I_i_*(*h*) is the intensity of an individual measurement of the reflection, and 〈*I*(*h*)〉 is the mean intensity of the reflection.

*^b^ R*_cryst_ = Σ*_h_*‖*F*_obs_| − |*F*_calc_‖/Σ*_h_*|*F*_obs_|, where *F*_obs_ and *F*_calc_ are the observed and calculated structure factor amplitudes, respectively.

*^c^ R*_free_ was calculated as *R*_cryst_ using 5.0% of randomly selected unique reflections that were omitted from the structure refinement.

**FIGURE 2.**
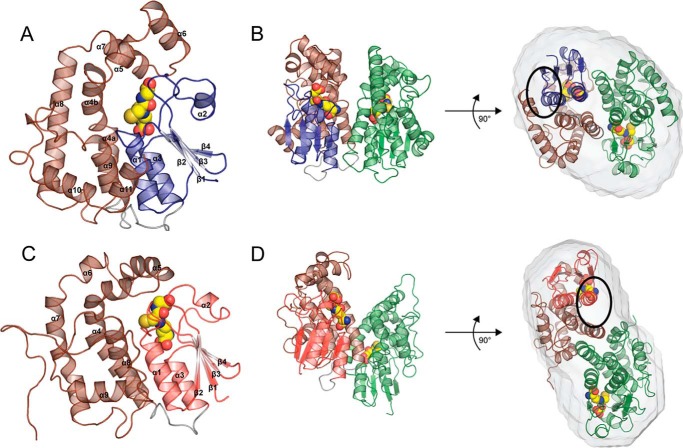
**LigE and LigF structures.** *A*, schematic representation of LigF, including the N-terminal thioredoxin domain (*blue*), the C-terminal α-helical domain (*brown*), and the short linker (*gray*). Bound GSH is shown as *yellow spheres. B*, schematic representation of the LigF dimer with the proposed substrate binding site (Fig. 5*A*) *circled. C*, schematic representation of LigE, including the N-terminal thioredoxin domain (*red*), the C-terminal α-helical domain (*brown*), and the short linker (*gray*). Bound GSH is shown as *yellow spheres. D*, schematic representation of the dimer of LigE with the proposed binding site (Fig. 5*B*) *circled*.

Consistent with their classification as GST enzymes, LigE and LigF each adopt the canonical GST domain fold with an N-terminal thioredoxin domain (residues 1–82 and 1–76, respectively) and a C-terminal α-helical domain (residues 93–255 and 93–242, respectively) connected by a short linker (residues 83–92 and 77–92, respectively) (Fig. 2). In both LigE and LigF, the thioredoxin domain comprises four β-strands and three α-helices following the topology β1α1β2α2β3β4α3. The loop between β1 and α1 is longer in LigE than in LigF and occupies the space between the thioredoxin domain and the α-helical domain, whereas in LigF, this loop is moved away from the domain interface toward the surface of the thioredoxin domain. The loop between β2 and α2 is longer in LigF than in LigE, but both interact with the α-helical domain on the protein face opposite the linker (Fig. 2). The C-terminal domains of both LigE and LigF are composed of six and eight α-helices, respectively. The root mean square deviation between the C-α locations of monomers of LigE and LigF is 4.42 Å, indicating that, although they catalyze very similar reactions, the enzymes display significant structural differences.

**FIGURE 3.**
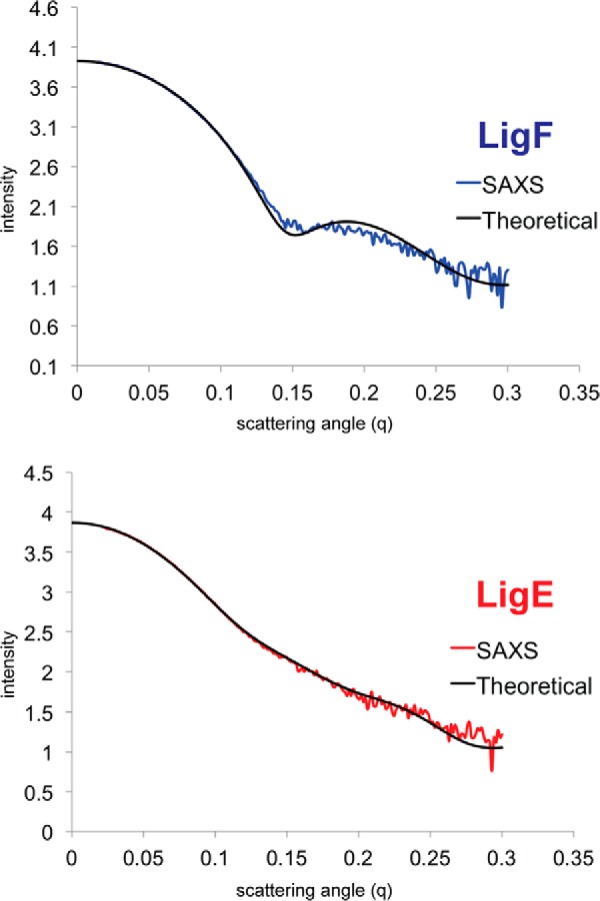
**Theoretical and experimental small angle x-ray scattering scatter curves.** The scattering angle (*q*) *versus* the intensity of the scattering plots shows the experimentally observed data and the theoretical scattering determined using CRYSOL from the x-ray structures of the dimers. LigF is shown at the *top* in *blue*, and LigE is shown at the *bottom* in *red*.

Biochemical and small angle x-ray scattering data suggest that both LigE and LigF exist as dimers in solution, and these dimers, related by 2-fold symmetry, can be seen in the respective crystal structures. The dimer interface accounts for 1,066 and 1,092 Å^2^ of buried surface area in LigE and LigF, respectively (PISA European Bioinformatics Institute) (54). The overall dimeric shapes of both LigF and LigE were confirmed using small angle x-ray scattering on both the truncated and full-length proteins. The protein envelopes determined by *ab initio* modeling align well with the crystal structures of both proteins (Fig. 2). The theoretical scattering curves predicted from the x-ray structures match well with the experimentally determined scattering curves with a χ value of 2.4 and 1.4 for LigF and LigE, respectively (Fig. 3).

**FIGURE 4.**
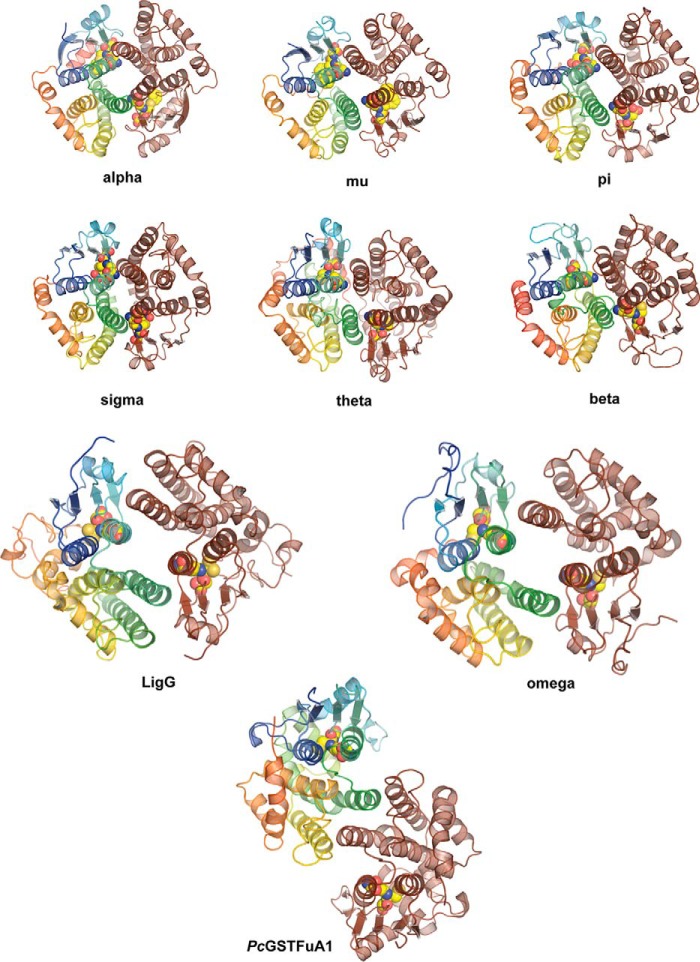
**Representative cytosolic GST dimer forms.** Representatives from several GST classes are shown, in which one molecule of the dimer is shown in *brown*, the second molecule of the dimer is shown in *rainbow colors* (N terminus in *blue* to C terminus in *red*), and the bound glutathione or glutathione analog is shown as *yellow spheres*. The Alpha (Protein Data Bank entry 1GUH; human GST A1-1), Mu (2GST; rat), Pi (2GSR; pGST P1-1 from pig), Sigma (1GSQ; squid), Theta (1LJR; human hGST T2-2), Beta (2PMT; bacterial GST from *P. mirabilis*), Omega (3LFL; human GST Omega-1), and LigG (4G10; *Sphingobium* sp. SYK-6) dimers show variations on the α3/α4 canonical four-helix bundle dimer structure, whereas the GSTFuA structure from *P. chrysosporium* shows a non-canonical dimer formed via interaction between α4 and the C-terminal domain of the second molecule of the dimer.

The LigF dimer forms via interactions between helices α3 and α4, in the thioredoxin and C-terminal domains, respectively, of each monomer, forming a four-helix bundle. The dimer interface is largely polar, lacking the traditional lock-and-key motif or hydrophobic surface common in other GST dimers, specifically the Alpha, Pi, and Mu classes (14, 15). The LigF dimer more closes matches those of the Beta or Theta class, which, like LigF, lack a hydrophobic lock-and-key motif, and there is no open V-shape to the dimer interface (14). Although the arrangement and characterization of the dimer forms in GST structures differ within and between classes, most are canonically anchored through contacts between α3 (or the final helix of the thioredoxin domain) and α4 (the first helix in the C-terminal domain) (13, 15, 55, 56). Variability in the arrangement of secondary structural elements away from the α3/α4 four-helix bundle changes the total buried surface area of the various GST dimers as well as changing the architecture of the enzyme in the vicinity of the active site (57). Representative structures demonstrating the variability of dimer packing in GSTs are shown in Fig. 4. The Alpha (Protein Data Bank entry 1GUH, human GST A1-1), Mu (2GST, rat), Pi (2GSR, pGST P1-1 from pig), Sigma (1GSQ, squid), Theta (1LJR, human hGST T2-2), Beta (2PMT, bacterial GST from *Proteus mirabilis*), Omega (3LFL, human GST Omega-1), and LigG (4G10, *Sphingobium* sp. SYK-6) dimers show variations on the α3/α4 canonical 4-helix bundle dimer structure (58–65). In the LigE dimer, helix α4 of one monomer is interdigitated between α4 and α7 of the other monomer, and the entire dimer interface is contained within the α-helical domain. The dimer is anchored by a hydrophobic lock-and-key motif in which Phe-101 of each monomer is in a hydrophobic pocket formed in the second monomer. This motif is seen in several GST classes, including Alpha, Mu, and Phi, which display the more typical four-helix bundle dimer mode rather than the elongated dimer of LigE (57). This elongated atypical dimer form in a GST was first described for GSTFuA1 from *Phanerochaete chrysosporium* (Fig. 4) in the GSTFuA class of GSTs, of which LigE is a member (13, 18). An additional β-hairpin motif between α2 and β3 in GST5118 hinders the formation of the regular α3/α4 GST dimer;however, this β-hairpin is not present in LigE (13). An extended loop between α5 and α6 in the C-terminal domain, which protrudes above the normal α3/α4 packing site, may be responsible for the alternate dimer formation in LigE. LigG, a known GST lyase in the *Sphingobium* β-ether degradation pathway, is an Omega class GST with the canonical α3/α4 GST dimer with a wider opening in the C-terminal domain, allowing for a proposed substrate binding site on the same face as that in LigE (65).

**FIGURE 5.**
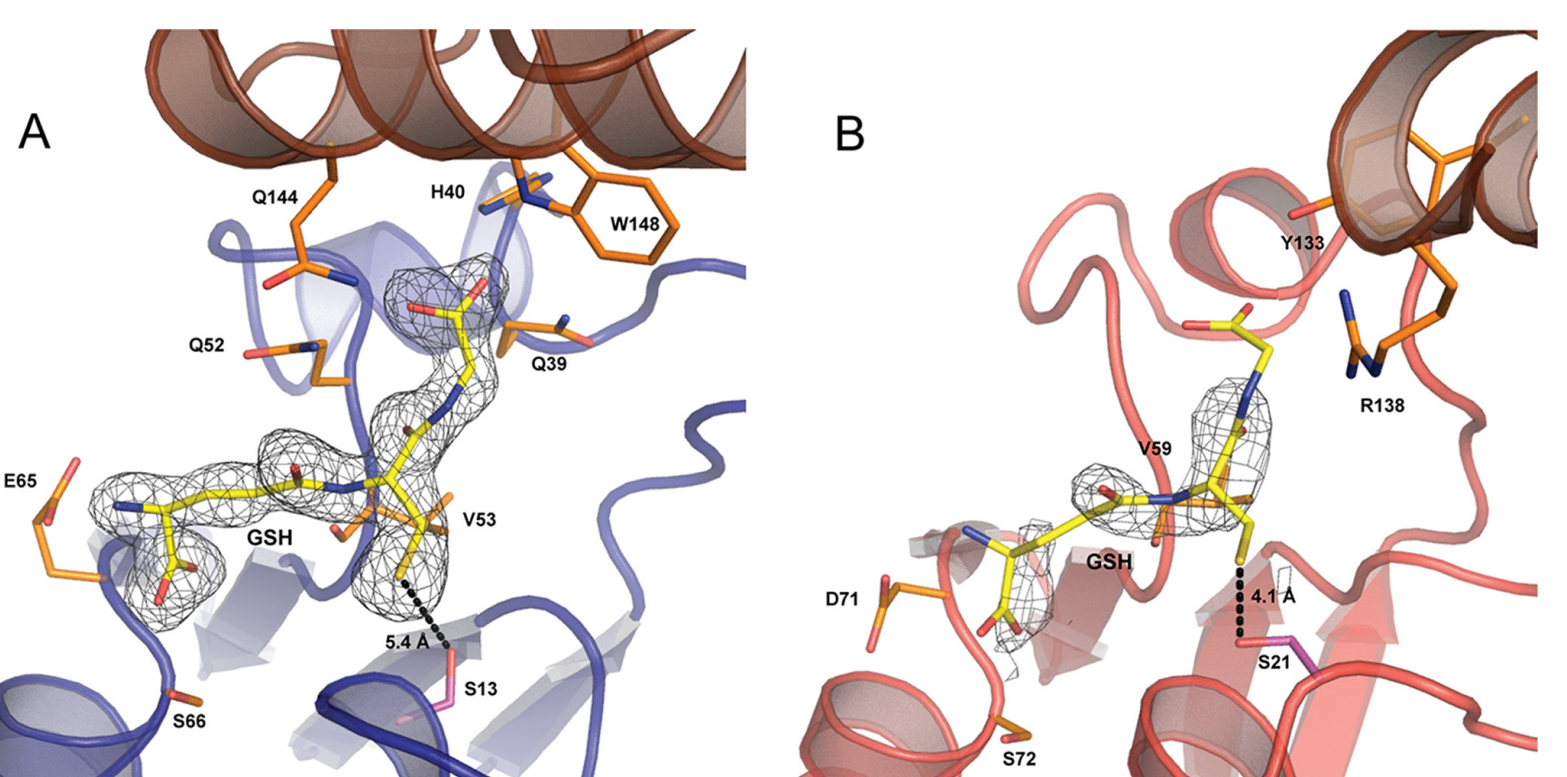
**Glutathione binding sites in LigF and LigE.** *A*, the GSH binding site in LigF is located in a cleft between the thioredoxin and α-helical domains. Density for the bound GSH (*yellow sticks*) is shown in *gray* contoured to 1.0σ (*CC* = 0.97). Residues interacting with the γ-glutamyl (Glu-65 and Ser-66), cysteinyl (Gln-52 and Val-53), and glycine (Gln-144, His-40, Trp-148, and Gln-39) residues of the bound GSH are shown as *orange sticks*. The distance between the GSH sulfur and the active site serine 13 (*purple sticks*) is 5.4 Å. *B*, the GSH binding in LigE is located on a surface-exposed face between the thioredoxin and α-helical domains. Density for the bound GSH (*yellow sticks*) from chain A of the model is shown in *gray* contoured to 1.0σ (*CC* = 0.71). Residues interacting with the γ-glutamyl (Asp-71 and Ser-72), cysteinyl (Val-59), and glycine (Arg-138 and Tyr-133) of the GSH are shown as *orange sticks*. The distance between the GSH sulfur and the active site serine 21 (*purple sticks*) is 4.1 Å.

The enzymatic active sites of these GST family members are often located in a cleft between the thioredoxin domain and the α-helical domain (15). Both the LigE and LigF enzymes contain the ββα motif required for anchoring GSH in the active site (56). In LigF, Glu-65 and Ser-66 located in the turn connecting β4 and α3, recognize the γ-glutamyl moiety of GSH as part of the ββα motif (Fig. 5*A*). Additionally, Gln-52 and the backbone of Val-53 interact with the cysteinyl moiety, whereas Gln-144, His-40, Tyr-148, and Gln-39 anchor the glycine residue of the active site GSH molecule. In LigE, Asp-71 and Ser-72, both located in the turn between β4 and α3, hydrogen-bond with the amino and carboxylate groups, respectively, of the γ-glutamyl residue of the GSH molecule (Fig. 5*B*). Additionally, the backbone of Val-59 interacts with the cysteinyl moiety, whereas weak hydrogen bonds are formed between the GSH glycine and Arg-138 and Tyr-133.

**FIGURE 6.**
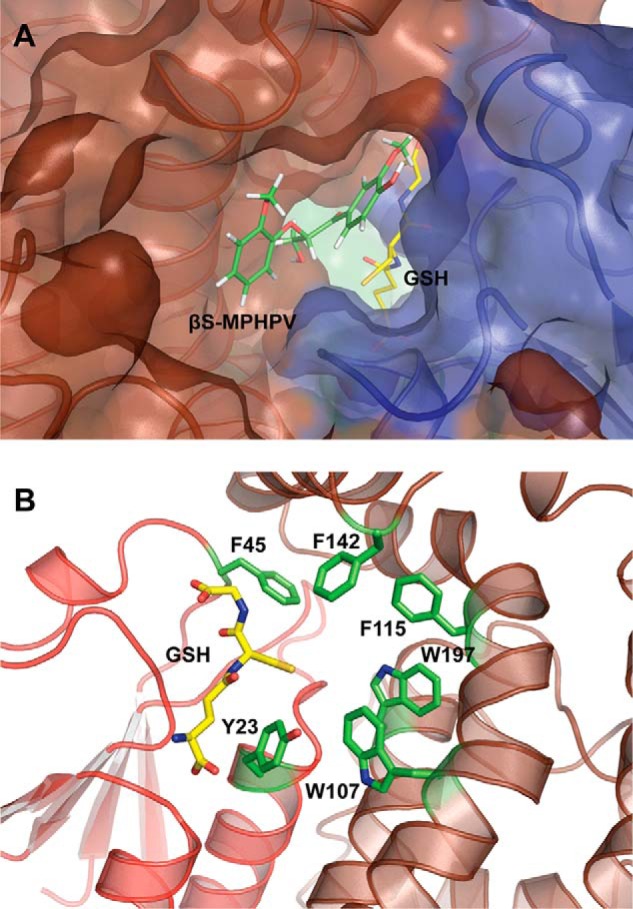
**Substrate binding sites in LigF and LigE.** *A*, model of ternary complex LigFΔ242-GSH·(β*S*)-MPHPV. Schematic representations are shown of the N-terminal thioredoxin domain (*blue*) and the C-terminal α-helical domain (*brown*) with the *circled region* from Fig. 2*B* detailed in a *transparent surface rendering*. The bound glutathione (*yellow*) and docked (β*S*)-MPHPV (*green*) are shown as *sticks. B*, proposed substrate binding surface in LigE. Schematic representations are shown for the LigE dimer, and the *circled region* from Fig. 2*D* is detailed, showing the hydrophobic aromatic substrate binding pocket formed by Phe-45, Phe-142, Phe-115, Trp-197, Trp-107, and Tyr-23 as *green sticks*.

Due to the occlusion of one face of the GSH binding pocket in LigF, we propose that the substrate binding site is located on the opposite face of the LigF monomer from the dimer interface (Fig. 2*B*, *black circle*). In the absence of a substrate-bound structure, SwissDock (47, 48) was used to generate a LigFΔ242-GSH·(β*S*)-MPHPV complex model (Fig. 6*A*) from the LigFΔ242-GSH structure and a molecular model of (β*S*)-MPHPV. The model supports our assignment of the substrate binding site. However, in LigE, this side of the GSH binding pocket is blocked by a number of loops, whereas the face of the GSH binding site shared with the dimer has been opened, due to the dimer rearrangement (Fig. 2*D*, *black circle*). Based on the binding site of GSH in LigE, we propose a potential location for the native substrate-binding site at the highly hydrophobic region consisting of residues Tyr^23^, Phe^45^, Trp^107^, Phe^115^, Phe^142^, and Trp^197^ (Fig. 6*B*). The aromatic rings of these hydrophobic residues are probably important in stacking interactions with the aromatic compounds from low molecular weight lignin derivative compounds.

The LigEΔ255-GSH and LigFΔ242-GSH structures revealed LigE Ser-21 (Fig. 5*B*) and LigF Ser-13 (Fig. 5*A*) as potential catalytic residues, based on their proximities to the thiol of the bound GSH. To further investigate the roles of LigE Ser-21 and LigF Ser-13 in β-etherase catalysis, variants LigE-S21A and LigFΔ242-S13A, in which serine residues were replaced with alanine, were expressed, purified, and tested for activity in the β-etherase assays.

##### Enzymatic Analysis and Mutagenesis

**FIGURE 7.**
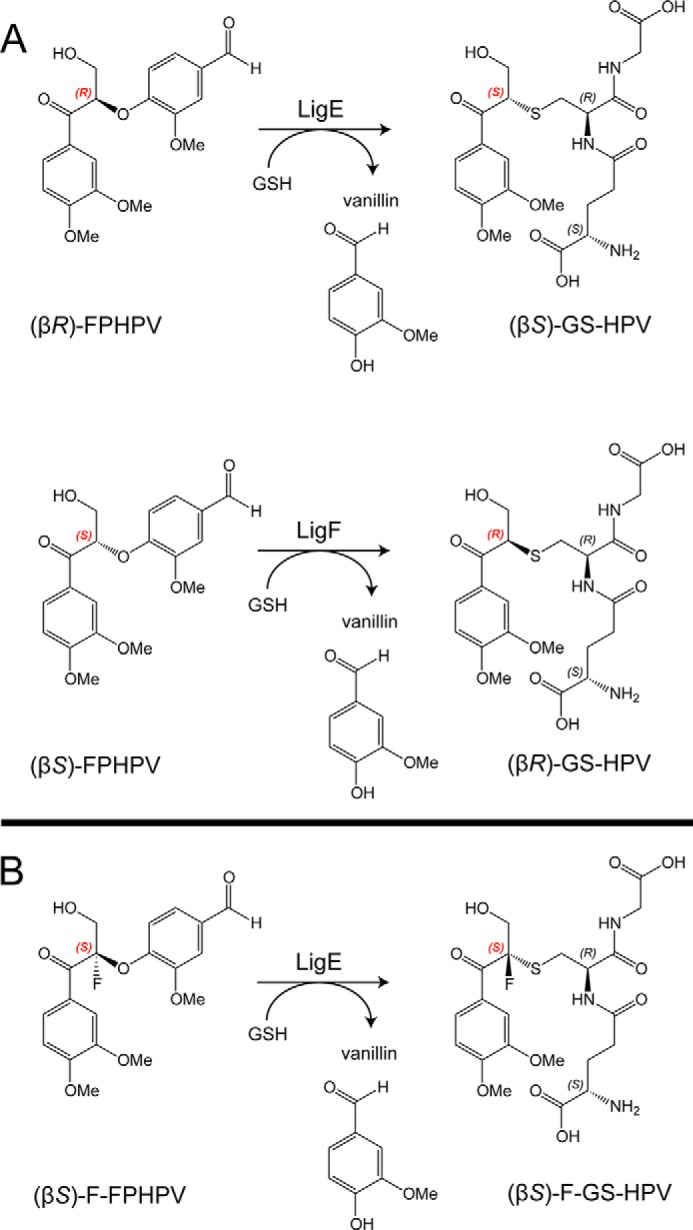
*A*, structure of an MPHPV analog substrate, FPHPV, that was used in the LigE- and LigF-catalyzed reactions, converting FPHPV to vanillin and GS-HVP. *B*, LigE-catalyzed β-ether elimination reaction with fluorinated model substrate (βS)-F-FPHPV, resulting in formation of vanillin and (β*S*)-F-GS-HVP.

**FIGURE 8.**
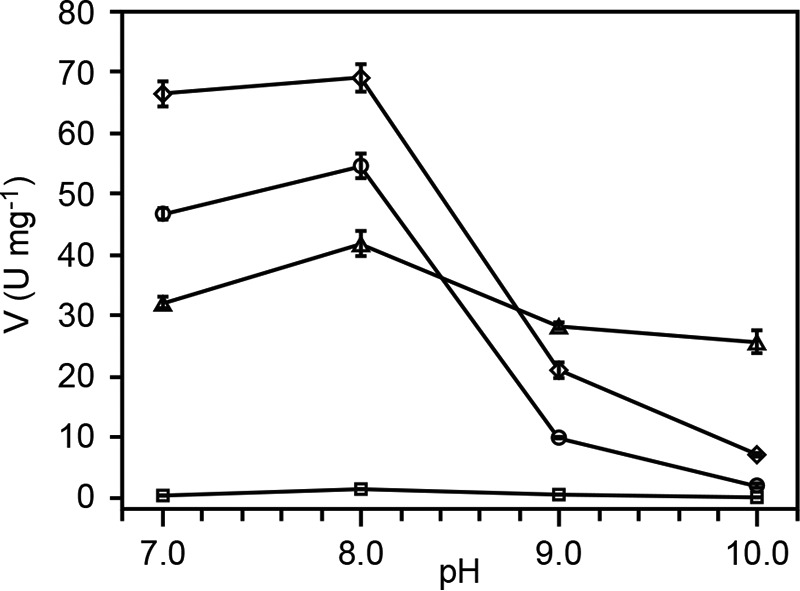
**LigE and LigF pH rate profile.** The effect of pH on β-etherase activities was determined for each enzyme, revealing that LigE (*triangles*), LigF (*circles*), LigFΔ242 (*diamonds*), and LigFΔ242-S13A (*squares*) have pH optima at pH 8.0. Plotted as a function of pH (*x axis*) are the specific enzymatic activities (*y axis*) of β-etherases with either (β*R*)-FPHPV (LigE) or (β*S*)-FPHPV (LigF, LigFΔ242, and LigFΔ242-S13A) as the assay substrate (1.5 mm initial concentration). *Error bars*, S.D. of triplicate measurements.

To analyze the enzymatic activities of the GSH-dependent β-etherase enzymes, FPHPV degradation rates were measured by the accumulation of vanillin, a monoaromatic product of FPHPV cleavage (Figs. 7 and 8). Whereas β-etherase catalysis with MPHPV results in the release of guaiacol (Fig. 1), vanillin is more easily detected by UV absorption, thus improving the sensitivity of the assays. In addition to LigE and LigF, we tested the rates of β-etherase catalysis for LigE variant LigE-S21A and two LigF variants, LigFΔ242 and LigFΔ242-S13A.

We found that LigE catalysis resulted in stereospecific (β*R*)-FPHPV cleavage, whereas LigF selectively degraded the (β*S*)-FPHPV enantiomer, as is consistent with previous reports (7, 9). The effect of pH on β-etherase activities was determined for each enzyme, revealing that LigE, LigF, LigFΔ242, and LigFΔ242-S13A have pH optima at pH 8.0 (Fig. 8). The activity of LigE was relatively unaffected by pH, whereas the activity of LigFΔ242 and LigFΔ242-S13A was significantly reduced above pH 8.0. The truncated LigFΔ242 exhibited higher rates of catalysis than full-length LigF at all pH values, indicating that the predicted disordered region in the C terminus may actually be inhibitory to β-etherase activity. The specific activities of LigE-S21A and LigFΔ242-S13A were 14% and <5% (Fig. 8 and Table 2) of the wild type and LigFΔ242, respectively, consistent with the structure-based predictions that these serine residues are involved in catalysis. Given the proximities of LigE Ser-21 and LigF Ser-13 hydroxyls to the GSH thiol (4.1 and 5.4 Å, respectively; Fig. 5) and because the specific activities of the β-etherases did not steadily increase as a function of increasing pH (Fig. 8), it is unlikely that these serine residues activate the GSH thiol for nucleophilic attack; rather, they act in GSH binding or thiol orientation or serve a different catalytic purpose.

**TABLE 2:** **LigE and LigF kinetic parameters** Kinetic parameters, determined from Michaelis-Menten curves for GSH-dependent β-etherases LigE, LigF, and their variants with substrates (β*R*)-MPHPV, (β*R*)-FPHPV, (β*S*)-F-FPHPV, and (β*S*)-FPHPV at pH 8.0. NDA, no detectable activity.

Enzyme	Substrate	*V*_max_*^a^*	% WT activity with (β*S*)-FPHPV*^b^*	*k*_cat_	*K_m_*	*k*_cat_/*K_m_*
		units mg^−*1*^	%	s^−*1*^	μ*m*	m*m*^−*1*^ s^−*1*^
LigE	(β*R*)-FPHPV	59.7 ± 1.2		31.9 ± 0.6	554 ± 16	57.6 ± 4.8
LigE-S21A	(β*R*)-MPHPV		13.5*^b^*			
LigF	(β*S*)-FPHPV	63.8 ± 0.4	100	31.9 ± 0.1	269 ± 1	118.4 ± 1.1
LigFΔ242	(β*S*)-FPHPV	69.3 ± 4.9*^a^*	108.7			
LigFΔ242-S13A	(β*S*)-FPHPV	1.5 ± 0.1*^a^*	2.3			
LigE	(β*S*)-F-FPHPV	0.02*^a^*				
LigF	(β*R*)-F-FPHPV	NDA				

*^a^* Where noted (*i.e.* in the absence of Michaelis-Menten curves), activity is reported as the velocity from assays in which the initial substrate concentration was 1.5 mm.

*^b^* Where noted, independent assays using substrate (β*R*)-MPHPV and either LigE or LigE-S21A as catalysts indicated that the LigE *V*_max_ was approximately 7-fold greater than that for LigE-S21A.

Because LigF Ser-13 was the only potential acid-base catalyst revealed in the active site of the LigFΔ242-GSH structure (Fig. 5*A*), we hypothesized that an S_N_2-type nucleophilic attack mechanism is responsible for catalysis in LigF. The LigFΔ242-GSH·(β*S*)-MPHPV complex model, generated using SwissDock (47, 48), revealed that the GSH thiolate is in the appropriate orientation for an S_N_2 attack relative to the substrate β-carbon.

The LigEΔ255-GSH structure revealed several potential catalytic residues in the active site, leaving open the possibility that the LigE β-etherase mechanism involves additional acid-base reactions. A substrate analog model compound, (β*S*)-fluoro-(1′-formyl-3′-methoxyphenoxy)-γ-hydroxypropioveratrone [(β*S*)-F-FPHPV] (Fig. 7), was used to test the possibility of a non-S_N_2 mechanism that would involve the deprotonation of the β-carbon of the substrate. (β*S*)-F-FPHPV and (β*R*)-FPHPV (despite their Cahn-Ingold-Prelog-derived *R*/*S* notations (66)) have the same enantiomeric configuration with respect to the orientation of their β-ether bonds and differ only in replacement of the hydrogen at the β-carbon in (β*R*)-FPHPV with a fluorine in (β*S*)-F-FPHPV, and this fluorine is predicted to prohibit deprotonation. We found that LigE catalyzed conversion of (β*S*)-F-FPHPV to vanillin and a glutathione-conjugated coproduct, albeit at a much lower velocity compared with cleavage of (β*R*)-FPHPV (Table 2), exactly as predicted based on the hypothesis that an S_N_2 catalytic mechanism would not involve deprotonation of the β-proton. Based on NMR analysis of the reaction products, we conclude that the LigE-catalyzed β-ether cleavage of (β*S*)-F-FPHPV resulted in formation of the expected glutathione-conjugated product, (β*S*)-F-GS-HPV. Although it is unclear why the reaction with (β*S*)-F-FPHPV was some 3 orders of magnitude slower than LigE-catalyzed cleavage of (β*R*)-FPHPV (Table 2), we hypothesize that the fluorine atom affects the β-ether bond angle and inhibits the approach of the thiolate ion for S_N_2 elimination. It is possible that these effects were even more pronounced in the active site of LigF, because LigF showed no detectable activity with the (β*R*)-F-FPHPV enantiomer.

## Discussion

The biocatalytic breakdown of lignin-derived compounds represents a potential source of aromatic products that would be valuable for the chemical, food, and pharmaceutical industries (2). In contrast to known fungal systems, the bacterium *Sphingobium* sp. strain SYK-6 possesses an enzymatic route to the breakdown of lignin-derived components that is stereospecific and independent of chemical mediators and requires common cellular cofactors, such as pyridine nucleotides and glutathione. These combined structural and biochemical studies of the β-aryl ether cleavage pathway enzymes provide insights into the features important for substrate and cofactor binding and catalysis. We propose that both LigE and LigF cleave β-ether-linked lignin dimer molecules via an S_N_2 nucleophilic attack on the β-carbon of the substrate that is consistent with previous results showing inversion of the chiral center at the β-carbon (9). Because LigE catalyzed the conversion of (β*S*)-F-FPHPV to (β*S*)-F-GS-HVP, we conclude that the LigE mechanism is unlikely to involve formation of an enzyme-substrate adduct and does not involve Cβ deprotonation or substrate enolization.

Although the sequences and x-ray crystal structures show a conserved serine in the active site of both LigE and LigF (serine 21 and 13, respectively) near the thiol of the bound glutathione (4.1 and 5.4 Å, respectively; Fig. 5), the serine is not essential for catalysis. In both LigE and LigF, mutation of the active site serine greatly reduced, but did not abolish, the enzymatic activity and did not shift the pH optimum, indicating that it may play a role other than deprotonation of the GSH thiol or perturbation of the apparent p*K_a_* of the bound glutathione. A conserved catalytic serine is a characteristic of the Theta class, Zeta class, and some bacterial GSTs (15), but there is evidence of GSTs from the bacteria *P. mirabilis*, *Ochrubactrum anthropi*, and *E. coli* in which this active site serine is not critical for catalytic activity (67–69). Based on the data presented here and support from previous studies, it is clear that although the active site serine is not responsible for the direct activation of the thiolate anion by deprotonation or perturbation of the p*K_a_* of the bound glutathione, it may be active in binding GSH in the active site, orienting the sulfhydryl group of GSH in the catalytic step, or stabilization of the transition state. Because GSH-dependent cleavage of these molecules does not occur readily *in vitro* in the absence of enzyme, it may be that the enzyme is able to stabilize the thiolate anion via a network of interactions within the active site or that the binding of the substrates in the optimal orientation and distance for the S_N_2 attack is sufficient for catalysis.

The structures of the LigE and LigF enzymes also highlight the nature of stereospecific control that is key to this pathway. These enzymes possess dramatically different structural arrangements within the monomers and different dimer interfaces, reflected in very different dimer shapes. As a result, the substrate binding surfaces of the two enzymes are on opposite faces of the thioredoxin domain and glutathione binding site. This observation means that if a substrate with the wrong stereochemistry were to bind, it would not be in the correct orientation with respect to the glutathione for catalysis, hence introducing stereospecificity. Due to the completely different geometry of the active site, there is no simple set of mutations that would switch substrate specificity or make each individual enzyme more promiscuous.

Based on structural properties, LigE is most similar to the fungal GSTFuA class (13), suggesting that the enzymes in this class are present in both prokaryotes and fungi. Other representatives in this class are from saprotrophic fungi, suggesting a functional connection among the members of the class (18). Although it has been suggested that LigF also belongs in the GSTFuA class (13), the dimer interface present in the structure is inconsistent with other members of the class. Based on our data, LigF is best placed in a new structural class closely related to GSTFuAs or as a fungal Ure2p-like GST based on structural similarities and function in saprotrophic organisms, although it does not strictly fit the class (70). Assignments to different GST family classes, combined with the structural and biochemical information presented here, suggest that LigE and LigF evolved to cleave unique stereoisomers of the aromatic dimers that are predicted to be found in plant lignins.

The detailed structural and biochemical characterization of LigE and LigF in this study and other members of the β-aryl etherase pathway reveal important new aspects of the enzyme mechanism and the determinants of substrate stereospecificity. Future enzyme engineering studies informed by these results may focus on optimizing the pathway for catalysis of specific lignin-derived compounds, formed as the byproducts of industrial biomass processing, into suitable products for use as, or precursors of, advanced biofuels and renewable chemicals.

## Author Contributions

K. E. H. designed experiments, produced protein, solved structure, analyzed LigF structure, performed all small angle x-ray scattering experiments, co-wrote the initial draft of manuscript, designed and compiled figures, and edited the manuscript; J. H. P. designed experiments, produced protein, solved structure, analyzed LigE structures, co-wrote initial draft of manuscript, and edited. D. L. G. designed experiments, synthesized substrates, cloned genes, expressed protein, performed enzymatic assays, co-wrote the initial draft of the manuscript, and edited the manuscript; R. A. H. designed experiments, cloned genes, expressed protein, performed enzymatic assays, and edited the manuscript; R. P. M. performed crystallographic data collection; C. B. designed experiments, assisted in construct design and crystallomics, and coordinated x-ray data collection; K. D. synthesized substrates; K. C. H. produced protein; D. R. N. designed experiments, led enzymology on LigF, provided substrates, and edited the manuscript; B. A. S. provided research direction, contributed lignin-specific and ligninolytic enzyme expertise, and edited the manuscript; K. L. S. provided research direction, contributed lignin-specific and ligninolytic enzyme expertise, and edited the manuscript; J. R. provided research direction, contributed lignin-specific expertise, and edited the manuscript; T. J. D. provided research direction, contributed microbiological specific expertise, and edited the manuscript; P. D. A. provided research direction, contributed crystallographic and structural biology expertise, and edited the manuscript; G. N. P. provided research direction, contributed crystallographic and structural enzymology expertise, and edited the manuscript.

## Supplementary Material

Supplemental Data

## Footnotes

5 The abbreviations used are:

GGE: guaiacylglycerol-β-guaiacyl ether
MPHPV: β-(3′-methoxyphenoxy)-γ-hydroxypropiovanillone
GS-HPV: β-glutathionyl-γ-hydroxypropiovanillone
CPD: cysteine protease domain
FPHPV: β-(1′-formyl-3′-methoxyphenoxy)-γ-hydroxypropioveratrone
F-FPHPV: fluoro-(1′-formyl-3′-methoxyphenoxy)-γ-hydroxypropioveratrone.
