# Supplementary material for: Structural Basis of Stereospecificity in the Bacterial Enzymatic Cleavage of β-Aryl Ether Bonds in Lignin
Source: J Biol Chem. 2015 Dec 4;291(10):5234–46. doi: 10.1074/jbc.M115.694307 (PMC4777856; doi:10.1074/jbc.M115.694307)
Supplement: Supplemental Data [file supp_291_10_5234__index.html]

Structural basis of stereospecificity in the bacterial enzymatic cleavage of β-aryl ether bonds in lignin — Structural basis of stereospecificity in the bacterial enzymatic cleavage of β-aryl ether bonds in lignin — Structural Basis of Stereospecificity in the Bacterial Enzymatic Cleavage of β-Aryl Ether Bonds in Lignin — Sphingobium LigE and LigF Crystal Structures — Supplemental Data 

# Structural Basis of Stereospecificity in the Bacterial Enzymatic Cleavage of β-Aryl Ether Bonds in Lignin

## Supplemental Data

- Supplement (.pdf, 1.2 MB) - Supplemental figures and data
