# Supplementary material for: Structural Basis of Stereospecificity in the Bacterial Enzymatic Cleavage of β-Aryl Ether Bonds in Lignin
Source: J Biol Chem. 2015 Dec 4;291(10):5234–46. doi: 10.1074/jbc.M115.694307 (PMC4777856; doi:10.1074/jbc.M115.694307)

Structural basis of stereospecificity in the bacterial biodegradation of β-aryl ether bonds found in lignin

Kate E. Helmich[1,2#], Jose Henrique Pereira[3,4#], Daniel L. Gall[2,5#], Richard A. Heins[3,6], Ryan P. McAndrew[3,4], Craig Bingman[1], Kai Deng[3,6], Keefe C. Holland[3,6], , Daniel R. Noguera[2,5], Blake A. Simmons[3,6], Kenneth L. Sale[3,6], John Ralph[1,2], Timothy J. Donohue[2,7], Paul D. Adams[3,4,8], George N. Phillips Jr.[9]

[1] Department of Biochemistry, University of Wisconsin, Madison, WI 53706

[2] U.S. Department of Energy Great Lakes Bioenergy Research Center, Wisconsin Energy Institute, University of Wisconsin, Madison, WI, 53726

[3] Joint BioEnergy Institute, Emeryville, CA 94608, USA

[4] Physical Biosciences Division, Lawrence Berkeley National Laboratory, Berkeley, CA 94720, USA

[5] Department of Civil & Environmental Engineering, University of Wisconsin, Madison, WI, 53706

[6] Biological and Engineering Sciences Center, Sandia National Laboratories, Livermore, CA, USA 94551 USA

[7] Department of Bacteriology, University of Wisconsin, Madison, WI, 53706

[8] Department of Bioengineering, University of California Berkeley, CA 94720, USA

[9] Department of Biochemistry and Cell Biology, Rice University, Houston, TX

[#]These authors contributed equally to this work.

* Correspondence should be addressed to Timothy J. Donohue (email), George N. Phillips, Jr. (*georgep@rice.edu*) or Paul D. Adams (*pdadams@lbl.gov*)

SUPPLEMENTARY INFORMATION – CHEMICAL SYNTHESES AND NMR DATA

CHEMICAL SYNTHESES:

General. Reagents and chemicals were purchased from Sigma-Aldrich. ^{1}H and ^{13}C NMR spectra were recorded on a Bruker Biospin (Billerica, MA) AVANCE 700 MHz spectrometer fitted with a cryogenically cooled 5-mm TXI gradient probe with inverse geometry (proton coils closest to the sample). ^{19}F NMR spectra were recorded on a Bruker Biospin DMX 400 MHz spectrometer. Chemical shifts are reported in parts per million (ppm). *J* values are recorded in Hz. The central NMR solvent peaks were used as internal references (δ_H: 2.05 ppm and δ_C: 29.8 ppm for acetone-d_6; δ_H: 4.79 ppm for (HDO in) D_2O (1,2). Carbon and proton assignments reported for all compounds in the attached spectra were determined via the aid of 2D COSY, HSQC, and HMBC NMR spectra. Merck-EMD Millipore aluminum-backed Silica Gel 60 F_{254} normal-phase thin-layer chromatography plates were used for small-scale separation of organic compounds using a mixture of hexane and ethyl acetate as the mobile solvent.

Syntheses of β-(1'-formyl-3'-methoxyphenoxy)-acetoveratrone (FPAV, **Supplementary Figure 3**) and racemic β-(1'-formyl-3'-methoxyphenoxy)-γ-hydroxypropioveratrone (FPHPV) were carried out as described previously (3,4) according to the method of Adler and Eriksoo (5,6). Biotage KP-Sil silica gel was used for preparative separations of organic compounds by flash chromatography using a CombiFlash R_f delivery module using a mixture of hexane and ethyl acetate as the mobile phase.

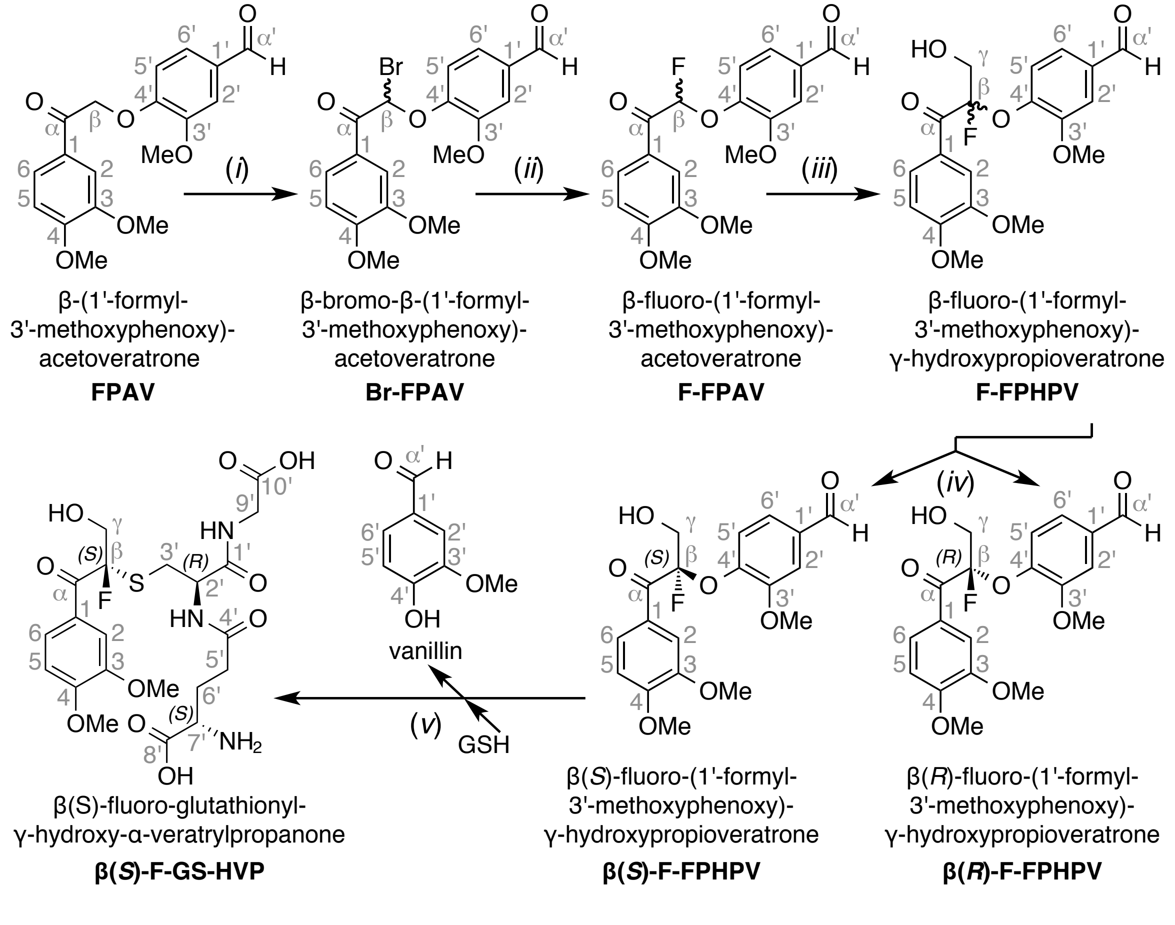

Supplementary Figure 3. Scheme for organic synthesis of (*i*) racemic β-bromo-(1'-formyl-3'-methoxyphenoxy)-acetoveratrone (Br-FPAV), (*ii*) racemic β-fluoro-(1'-formyl-3'-methoxyphenoxy)-acetoveratrone (F-FPAV), (*iii*) racemic β-fluoro-(1'-formyl-3'-methoxyphenoxy)-γ-hydroxypropioveratrone (F-FPHPV), (*iv*) chiral chromatographic separation of enantiomers β(*S*)-F-FPHPV and β(*R*)-F-FPHPV, and (*v*) enzymatic synthesis of β(*S*)-fluoro-glutathionyl-γ-hydroxy-α-veratrylpropanone (β(*S*)-F-GS-HVP). Reagents and conditions: (*i*) pyridinium tribromide, EtOAc, 90 min, 68%; (*ii*) Ag(I)F, acetonitrile, 18 h, 90%; (*iii*) formaldehyde, K_2CO_3, 1,4-dioxane, 18 h, 23%; (*iv*) chiral chromatography, ethanol/hexane; (*v*) LigE, glutathione, 25 mM Tris in H_2O, pH 8.0, 18 h, C_{18} chromatography, H_2O/methanol.

Synthesis of *racem*-β-bromo-(1'-formyl-3'-methoxyphenoxy)-acetoveratrone (Br-FPAV), Supplementary Figure 8(*i*). Synthetic FPAV served as the starting material in the synthesis of *racem*-β-bromo-(1'-formyl-3'-methoxyphenoxy)-acetoveratrone (Br-FPAV, **Supplementary Figure 8(*i*)**). A solution of ethyl acetate (100 mL), FPAV (2.5 g, 7.6 mmol), and pyridinium tribromide (2.9 g, 9.1 mmol) was prepared in a 250-mL round-bottom flask with stirring. After 90 min, the reaction mixture was washed three times with saturated Na_2CO_3, once with H_2O, and once with brine. The organic layer was then dried over $MgSO_4$ and the solvent was evaporated *in vacuo*, and the residual oil was identified as Br-FPAV (2.1 g, 68% yield).

***racem*-β-bromo-(1'-formyl-3'-methoxyphenoxy)-acetoveratrone (Br-FPAV):**

^{1}H NMR (700 MHz, acetone-d_6) δ 10.00 (s, 1H, Hα′); 8.00 (dd, 1H, $J_{6\text{-}5}$ = 8.5 Hz, $J_{6\text{-}2}$ = 2.0 Hz, H6); 7.77 (d, 1H, $J_{2\text{-}6}$ = 2.0 Hz, H2); 7.68 (s, 1H, Hβ); 7.67 – 7.65 (m, 2H, H6′/H5′); 7.61 (d, 1H, $J_{2'\text{-}6'}$ = 1.7 Hz, H2′); 7.14 (d, 1H, $J_{5'\text{-}6'}$ = 8.5 Hz, H5); 3.97 (s, 3H, 3′-OMe); 3.95 (s, 3H, 4-OMe); 3.92 (s, 3H, 3-OMe).

^{13}C NMR (176 MHz, acetone-d_6) δ 191.7 (Cα′); 186.4 (Cα); 155.6 (C4); 152.2 (C3′); 150.2 (C3); 149.3 (C4′); 134.9 (C1′); 125.6 (C1); 125.4 (C6); 125.0 (C6′); 119.3 (C5′); 112.7 (C2); 112.3 (C2′); 111.6 (C5); 82.6 (Cβ); 56.6 (3′-OMe); 56.3 (4-OMe); 56.1 (3-OMe).

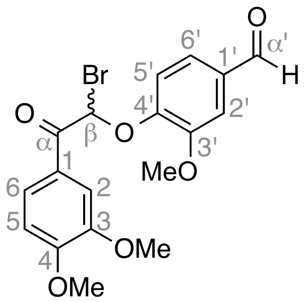

Synthesis of *racem*-β-fluoro-(1'-formyl-3'-methoxyphenoxy)-acetoveratrone (F-FPAV), Supplementary Figure 8(*ii*). To a magnetically stirred solution of Br-FPAV (2.0 g, 5.0 mmol) dissolved in anhydrous acetonitrile (50 mL) in a 250-mL round-bottom flask maintained under an inert atmosphere at 60 °C, silver (I) fluoride (Ag(I)F) was added (3.2 g, 25.0 mmol). After 18 h, inorganics (7) were removed by filtration and the filtrate was evaporated *in vacuo*. The resulting residue was taken up with ethyl acetate and washed once with aqueous NH_4Cl, three times with H_2O, and once with brine. The organic layer was then dried over $MgSO_4$ and the solvent was again evaporated *in vacuo*. The product oil was further purified by flash chromatography using a mobile phase of Hexane/EtOAc = 1/1 (R_f = 0.36). The product was then dissolved in hot ethyl acetate, hexane was added slowly and the mixture allowed to cool, affording crystalline *racem*-β-fluoro-(1'-formyl-3'-methoxyphenoxy)-acetoveratrone (F-FPAV, 1.6 g, 90% yield).

racem-β-fluoro-(1'-formyl-3'-methoxyphenoxy)-acetoveratrone (F-FPAV):

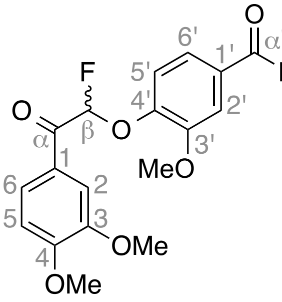

^{1}H NMR (700 MHz, acetone-d_6) δ 9.99 (s, 1H, Hα′); 7.91 (dd, 1H, $J_{6\text{-}5}$ = 8.5 Hz, $J_{6\text{-}2}$ = 1.8 Hz, H6); 7.67 (d, 1H, $J_{2\text{-}6}$ = 1.8 Hz, H2); 7.63 (dd, 1H, $J_{6'\text{-}5'}$ = 8.2 Hz, $J_{6'\text{-}2'}$ = 1.8 Hz, H6′); 7.61 (d, 1H, $J_{2'\text{-}6'}$ = 1.8 Hz, H2′); 7.55 (d, 1H, $J_{5\text{-}6}$ = 8.2 Hz, H5′); 7.15 (d, 1H, $J_{5'\text{-}6'}$ = 8.5 Hz, H5); 6.88 (d, 1H, $J_{\beta\text{-}\beta F}$ = 59.6 Hz, Hβ); 3.96 (s, 3H, 3′-OMe); 3.94 (s, 3H, 4-OMe); 3.90 (s, 3H, 3-OMe).

^{13}C NMR (176 MHz, acetone-d_6) δ 191.6 (Cα′); 187.4 (d, $J_{\alpha\text{-}\beta F}$ = 25.7 Hz, Cα); 155.8 (C4); 151.8 (C3′); 150.3 (C3); 150.1 (C4′); 134.8 (C1′); 126.4 (C1); 125.7 (C6); 125.2 (C6′); 119.5 (C5′); 112.3 (C2′); 112.2 (C2); 111.6 (C5); 106.1 (d, $J_{\beta\text{-}\beta F}$ = 232.9 Hz, Cβ); 56.5 (3′-OMe); 56.3 (4-OMe); 56.1 (3-OMe).

^{19}F NMR (376.5 MHz, acetone-d_6) δ -132.3 (d, $J_{\beta F\text{-}\beta H}$ = 59.6 Hz, Fβ).

Synthesis of _racem_-β-fluoro-(1'-formyl-3'-methoxyphenoxy)-γ-hydroxypropioveratrone (F-FPHPV) racemate, Supplementary Figure 8 (_iii–iv_). To a magnetically stirred solution of 1,4-dioxane (40 mL), F-FPAV (0.8 g, 2.3 mmol), and formaldehyde (136 mg, 4.5 mmol, 340 μL of 37% formaldehyde in H_2O) in a 100-mL round-bottom flask, anhydrous K_2CO_3 (0.4 g, 22.7 mmol) was added and the reaction was set to 60 °C. After 18 h, the reaction was cooled to room temperature, carbonates were removed by filtration, and 1,4-dioxane was evaporated *in vacuo*. The product was dissolved in ethyl acetate and washed three times with H_2O and once with brine. The organic layer was dried over $MgSO_4$ and the solvent evaporated *in vacuo*. The residue was further purified by flash chromatography using a mobile phase of Hexane/EtOAc = 1/3 (R_f = 0.19). Racemic β-fluoro-(1'-formyl-3'-methoxyphenoxy)-γ-hydroxypropioveratrone (F-FPHPV) was recovered from chromatographic fractions as an oil (0.2 g, 23% yield).

The chiral enantiomers of racemate F-FPHPV (*i.e.*, β(*S*)-F-FPHPV and β(*R*)-F-FPHPV) were purified via chiral chromatography where racemic F-FPHPV (5 mg, 13.2 mmol) was dissolved in ethanol (5 mL) and injected into a Beckman 125NM solvent module HPLC system equipped with a Diacel Chemical Industries CHIRALPAK AY-H column (10 by 250 mm) and Beckman 168 UV detector. A mixture of ethanol and hexane was used as the mobile phase at a flow rate of 2.0 mL $min.^{-1}$ The ethanol fraction of the total flow (with hexane as the remainder) was adjusted over a gradient as follows: 0–10 min, 10% ethanol; 10–60 min, gradient from 10-40% ethanol; 60–71 min, 40% ethanol; 71–72 min, gradient from 40-10% ethanol; 72–80 min, 10% ethanol. β(*S*)-F-FPHPV and β(*R*)-F-FPHPV eluted from the column after retention times (t_R) of 46.1 min (R_f = 0.42) and 53.9 min (R_f = 0.36), respectively, and their fractions were collected, pooled, and solvents were dried *in vacuo*.

racem-β-fluoro-(1'-formyl-3'-methoxyphenoxy)-γ-hydroxypropioveratrone (F-FPHPV):

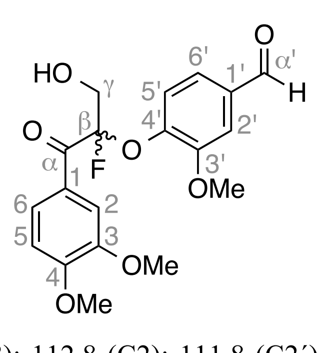

^{1}H NMR (700 MHz, acetone-d_6) δ 9.85 (s, 1H, Hα′); 7.93 (dd, 1H, $J_{6\text{-}5}$ = 8.6 Hz, $J_{6\text{-}2}$ = 2.0 Hz, H6); 7.64 (d, 1H, $J_{2\text{-}6}$ = 2.0 Hz, H2); 7.48 (d, 1H, $J_{2'\text{-}6'}$ = 1.8 Hz, H2′); 7.41 (dd, 1H, $J_{6'\text{-}5'}$ = 8.3 Hz, $J_{6'\text{-}2'}$ = 1.8 Hz, H6′); 7.28 (d, 1H, $J_{5\text{-}6}$ = 8.3 Hz, H5′); 7.04 (d, 1H, $J_{5'\text{-}6'}$ = 8.6 Hz, H5); 4.81 (dd, 1H, $J_{\gamma OH\text{-}\gamma a}$ = 7.6 Hz, $J_{\gamma OH\text{-}\gamma b}$ = 6.0 Hz, γ-OH); 4.27 (ddd, 1H, $J_{\gamma a\text{-}\beta F}$ = 23.2 Hz, $J_{\gamma a\text{-}\gamma b}$ = 12.4 Hz, $J_{\gamma a\text{-}\gamma OH}$ = 7.6 Hz, $H\gamma_a$); 4.19 (ddd, 1H, $J_{\gamma b\text{-}\beta F}$ = 12.5 Hz, $J_{\gamma b\text{-}\gamma a}$ = 12.4 Hz, $J_{\gamma b\text{-}\gamma OH}$ = 6.1 Hz, $H\gamma_b$); 3.97 (s, 3H, 3′-OMe); 3.89 (s, 3H, 4-OMe); 3.85 (s, 3H, 3-OMe).

^{13}C NMR (176 MHz, acetone-d_6) δ 191.4 (Cα′); 190.3 (d, $J_{\alpha\text{-}\beta F}$ = 27.6 Hz, Cα); 155.3 (C4); 151.6 (C3′); 149.8 (C3); 148.7 (C4′); 134.0 (C1′); 127.2 (C1); 125.6 (C6); 125.1 (C6′); 119.3 (C5′); 114.3 (d, $J_{\beta\text{-}\beta F}$ = 241.8 Hz, Cβ); 112.8 (C2); 111.8 (C2′); 111.4 (C5); 66.2 (d, $J_{\gamma\text{-}\beta F}$ = 28.5 Hz Cγ); 56.4 (3′-OMe); 56.2 (4-OMe); 56.0 (3-OMe).

^{19}F NMR (376.5 MHz, acetone-d_6) δ -117.7 (dd, $J_{\beta F\text{-}\gamma Ha}$ = 23.2 Hz, $J_{\beta F\text{-}\gamma Hb}$ = 12.5 Hz, Fβ).

Enzymatic synthesis of β(*S*)-fluoro-glutathionyl-γ-hydroxy-α-veratrylpropanone (β(*S*)-F-GS-HVP), Supplementary Figure 8(*v*). *In vitro* enzymatic synthesis with β-etherase LigE (360 μg mL^{-1}) as a catalyst was conducted in 4 mL of an aqueous assay buffer [1.5 mM β(*S*)-F-FPHPV, 25 mM Tris, 2.5% DMSO, 5 mM glutathione (GSH), pH 8.0]. Reagents were added as follows: 2.3 mg β(*S*)-F-FPHPV, 100 μL DMSO, 3500 μL of 25.6 mM Tris (pH 9.0), 200 μL of 100 mM GSH in 25.6 mM Tris, 200 μL of a 7.2 μg μL^{-1} stock of LigE. Prior to addition of LigE, the pH of the assay mixture was measured at pH 8.0 using pH paper. After 18 h, the 4 mL reaction was filtered through a 10,000 MWCO filter for the removal of protein and six ethyl acetate extractions were conducted for the removal of vanillin and residual β(*S*)-F-FPHPV. The aqueous fraction was then loaded onto a pre-packed Biotage KP-C_{18} (100 g) reversed phase column using a Beckman 125NM solvent delivery module equipped with a Beckman 168 UV detector. A mixture of water and methanol was used for the mobile phase at a flow rate of 10 mL/min. The proportions of the total flow made up by each buffer were adjusted over a gradient: 0–15 min, 0% methanol; 15–20 min, gradient from 0-100% methanol; 20–35 min, 100% methanol; 35–40 min, gradient from 100-0% methanol; 40–50 min, 0% methanol. After elution of the perceived GSH-conjugated reaction product (t_R = 31.0 min), fractions with UV absorption at 280 nm were collected, pooled, dried over a gentle stream of nitrogen gas overnight, and then analyzed by NMR. The ^{1}H, COSY, HSQC, and HMBC NMR spectra of the isolated product from the LigE-catalyzed reaction were consistent with the identity of β(*S*)-fluoro-glutathionyl-γ-hydroxy-α-veratrylpropanone (β(*S*)-F-GS-HVP**,** Figure S8A). Because β(*S*)-F-GS-HVP was recovered from the reaction in low quantity, a ^{13}C NMR spectrum could not be obtained directly. Rather, the ^{13}C NMR spectral data reported was determined from the ^{1}H, COSY, HSQC, and HMBC NMR spectra.

β(*S*)-fluoro-glutathionyl-γ-hydroxy-α-veratrylpropanone (β(*S*)-F-GS-HVP), from LigE:

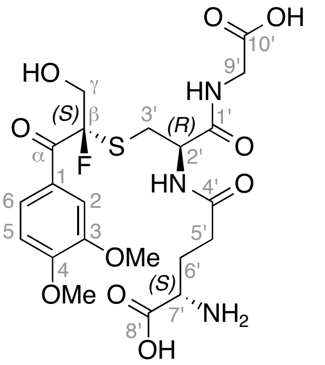

^{1}H NMR (700 MHz, D_2O) δ 7.89 (dd, 1H, $J_{6\text{-}5}$ = 8.6 Hz, $J_{6\text{-}2}$ = 2.0 Hz, H6); 7.59 (d, 1H, $J_{2\text{-}6}$ = 2.0 Hz, H2); 7.01 (d, 1 H, $J_{5\text{-}6}$ = 8.6 Hz, H5); 4.47 (dd, 1H, $J_{2'\text{-}3b'}$ = 8.1 Hz, $J_{2'\text{-}3a'}$ = 4.9 Hz, H2′); 4.21 (dd, 1H, $J_{\gamma a\text{-}\beta F}$ = 27.3 Hz, $J_{\gamma a\text{-}\gamma b}$ = 13.2 Hz, Hγ_a); 4.07 (dd, 1H, $J_{\gamma b\text{-}\beta F}$ = 15.8 Hz, $J_{\gamma b\text{-}\gamma a}$ = 13.2 Hz, Hγ_b); 3.84 (s, 3H, 4-OMe); 3.80 (s, 3H, 3-OMe); 3.62 (d, 1H, $J_{9a'\text{-}9b'}$ = 17.2 Hz, H9′$_a$); 3.60 (t, 1H, $J_{7'\text{-}6'}$ = 6.3 Hz, H7′); 3.48 (d, 1H, $J_{9b'\text{-}9a'}$ = 17.2 Hz, H9′$_b$); 3.05 (dd, 1H, $J_{3a'\text{-}3b'}$ = 14.1 Hz, $J_{3a'\text{-}2'}$ = 4.9 Hz, H3′$_a$); 2.90 (dd, 1H, $J_{3b'\text{-}3a'}$ = 14.1 Hz, $J_{3b'\text{-}2'}$ = 8.1 Hz, H3′$_b$); 2.34 – 2.28 (m, 2H, H5′$_{a/b}$); 2.00 – 1.94 (m, 2H, H6′$_{a/b}$).

^{13}C NMR (176 MHz, D_2O) δ 194.1 (d, $J_{\gamma\text{-}\beta F}$ = ~25 Hz, Cα); 176.1 (C10′); 175.2 (C4′); 174.6 (C8′); 171.5 (C1′); 154.0 (C4); 148.3 (C3); 127.0 (C1); 126.0 (C6); 112.6 (C2); 111.0 (C5); 107.7 (d, $J_{\beta\text{-}\beta F}$ = ~240 Hz, Cβ); 65.7 (d, $J_{\gamma\text{-}\beta F}$ = ~25 Hz, Cγ); 56.0 (4-OMe); 55.9 (3-OMe); 54.1 (C7′); 53.0 (C2′); 43.2 (C9′); 31.4 (C5′); 30.2 (C3′); 26.1 (C6′).

Enzymatic synthesis of β(*S*)-glutathionyl-γ-hydroxy-α-veratrylpropanone (β(*S*)-GS-HVP). With β(*R*)-FPHPV used as the reaction substrate, rather than β(*S*)-F-FPHPV, the same procedure was used to isolate the product β(*S*)-GS-HVP from a LigE-catalyzed reaction, as previously described (3).

β(*S*)-glutathionyl-γ-hydroxy-α-veratrylpropanone (β(*S*)-GS-HVP), from LigE:

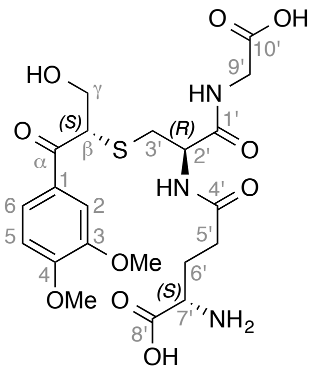

^{1}H NMR (700 MHz, D_2O) δ 7.68 (dd, 1H, $J_{6\text{-}5}$ = 8.4 Hz, $J_{6\text{-}2}$ = 2.0 Hz, H6); 7.48 (d, 1H, $J_{2\text{-}6}$ = 2.0 Hz, H2); 7.02 (d, 1 H, $J_{5\text{-}6}$ = 8.4 Hz, H5); 4.54 (dd, 1H, $J_{\beta\text{-}\gamma a}$ = 7.9, $J_{\beta\text{-}\gamma b}$ = 5.9 Hz, Hβ); 4.27 (dd, 1H, $J_{2'\text{-}3b'}$ = 8.8 Hz, $J_{2'\text{-}3a'}$ = 5.1 Hz, H2′); 3.98 (dd, 1H, $J_{\gamma a\text{-}\gamma b}$ = 11.6 Hz, $J_{\gamma a\text{-}\beta}$ = 7.9 Hz, Hγ_a); 3.84 (s, 3H, 4-OMe); 3.83 - 3.78 (m, 1H, Hγ_b); 3.80 (s, 3H, 3-OMe); 3.57 (d, 1H, $J_{9a'\text{-}9b'}$ = 17.3 Hz, H9′$_a$); 3.50 (d, 1H, $J_{9b'\text{-}9a'}$ = 17.3 Hz, H9′$_b$); 3.49 (t, 1H, $J_{7'\text{-}6'}$ = 6.4 Hz, H7′); 2.94 (dd, 1H, $J_{3a'\text{-}3b'}$ = 14.2 Hz, $J_{3a'\text{-}2'}$ = 5.1 Hz, H3′$_a$); 2.77 (dd, 1H, $J_{3b'\text{-}3a'}$ = 14.2 Hz, $J_{3b'\text{-}2'}$ = 8.8 Hz, H3′$_b$); 2.26 – 2.16 (m, 2H, H5′$_{a/b}$); 1.90 – 1.80 (m, 2H, H6′$_{a/b}$).

^{13}C NMR (176 MHz, D_2O) δ 198.4 (Cα); 176.8 (C10′); 175.5 (C4′); 175.4 (C8′); 172.1 (C1′); 154.4 (C4); 149.0 (C3); 128.9 (C1); 125.3 (C6); 111.8 (C2); 111.8 (C5); 61.6 (Cγ); 56.7 (4-OMe); 56.5 (3-OMe); 54.9 (C7′); 53.8 (C2′); 48.6 (Cβ); 44.0 (C9′); 32.2 (C5′); 32.1 (C3′); 27.3 (C6′).

SUPPLEMENTARY REFERENCES

1. Gottlieb, H. E., Kotlyar, V., and Nudelman, A. (1997) NMR chemical shifts of common laboratory solvents as trace impurities. *Journal of Organic Chemistry* **62**, 7512-7515.
2. Fulmer, G. R., Miller, A. J. M., Sherden, N. H., Gottlieb, H. E., Nudelman, A., Stoltz, B. M., Bercaw, J. E., and Goldberg, K. I. (2010) NMR chemical shifts of trace impurities: Common laboratory solvents, organics, and gases in deuterated solvents relevant to the organometallic chemist. *Organometallics* **29**, 2176-2179.
3. Gall, D. L., Kim, H., Lu, F., Donohue, T. J., Noguera, D. R., and Ralph, J. (2014) Stereochemical features of glutathione-dependent enzymes in the *Sphingobium* sp. strain SYK-6 β-aryl etherase pathway. *J Biol Chem* **289**, 8656-8667.
4. Gall, D. L., Ralph, J., Donohue, T. J., and Noguera, D. R. (2014) A group of sequence-related sphingomonad enzymes catalyzes cleavage of β-aryl ether linkages in lignin β-guaiacyl and β-syringyl ether dimers. *Environmental science & technology* **48**, 12454-12463.
5. Adler, E., and Eriksoo, E. (1955) Guaiacylglycerol and its β-guaiacyl ether. *Acta chemica Scandinavica* **9**, 341-342.
6. Landucci, L. L., Geddes, S. A., and Kirk, T. K. (1981) Synthesis of C-14-labeled 3-methoxy-4-hydroxy-α-(2-methoxyphenoxy)-β-hydroxypropiophenone, a lignin model-compound. *Holzforschung* **35**, 67-70.
7. Padmakshan, D., Bennett, S. A., Otting, G., and Easton, C. J. (2007) Stereocontrolled synthesis of (*S*)-γ-fluoroleucine. *Synlett*, 1083-1084.

SUPPLEMENTARY INFORMATION - ^{1}H AND ^{13}C NMR SPECTRA OF MODEL COMPOUNDS AND REACTION PRODUCTS

COMPOUND:	Source:[a]	Spectrum:	Solvent:[b]	Page:
racem-β-bromo-(1'-formyl-3'-methoxyphenoxy)-acetoveratrone (Br-FPAV)	synthetic	^{1}H	acetone-d_6	S-18
		^{13}C	acetone-d_6	S-19
racem-β-fluoro-(1'-formyl-3'-methoxyphenoxy)-acetoveratrone (F-FPAV)	synthetic	^{1}H	acetone-d_6	S-20
		^{13}C	acetone-d_6	S-21
		^{19}F	acetone-d_6	S-22
racem-β-fluoro-(1'-formyl-3'-methoxyphenoxy)-γ-hydroxypropio-veratrone (F-FPHPV)	synthetic	^{1}H	acetone-d_6	S-23
		^{13}C	acetone-d_6	S-24
		^{19}F	acetone-d_6	S-25
β(*S*)-fluoro-glutathionyl-γ-hydroxy-α-veratrylpropanone (β(*S*)-F-GS-HVP)	LigE	^{1}H	D_2O	S-26
β(*S*)-glutathionyl-γ-hydroxy-α-veratrylpropanone (β(*S*)-GS-HVP)	LigE	^{1}H	D_2O	S-27
		^{13}C	D_2O	S-28

[a] Compounds were obtained either via organic synthesis or from LigE β-etherase-catalyzed reaction products.
[b] ^{1}H, ^{13}C, and ^{19}F NMR spectra were analyzed in either acetone-d_6 or D_2O as NMR solvents.

***racem*-β-bromo-(1'-formyl-3'-methoxyphenoxy)-acetoveratrone (Br-FPAV)**

^{1}H NMR (700 MHz, acetone-d_6)

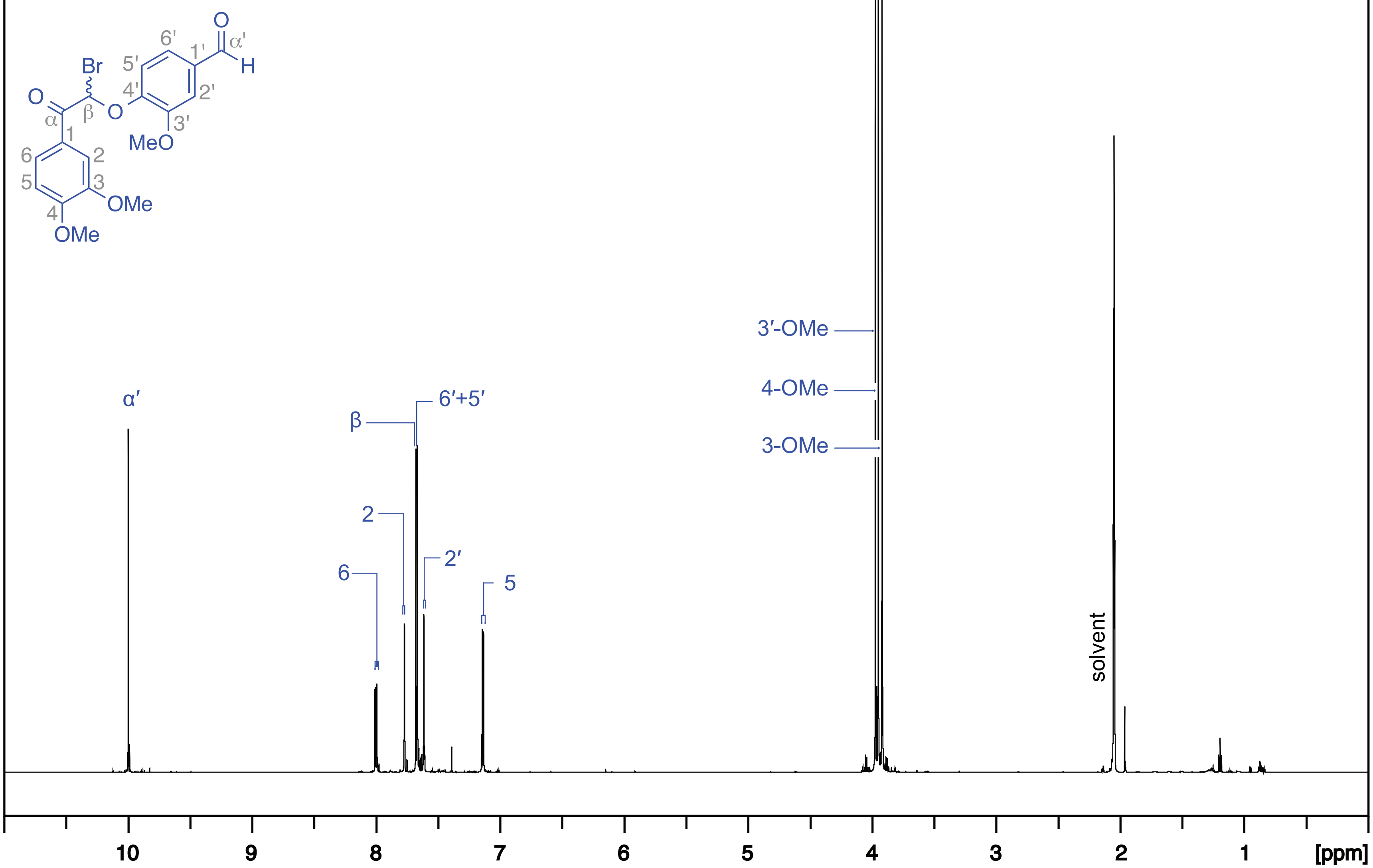

***racem*-β-bromo-(1'-formyl-3'-methoxyphenoxy)-acetoveratrone (Br-FPAV)**

^{13}C NMR (176 MHz, acetone-d_6)

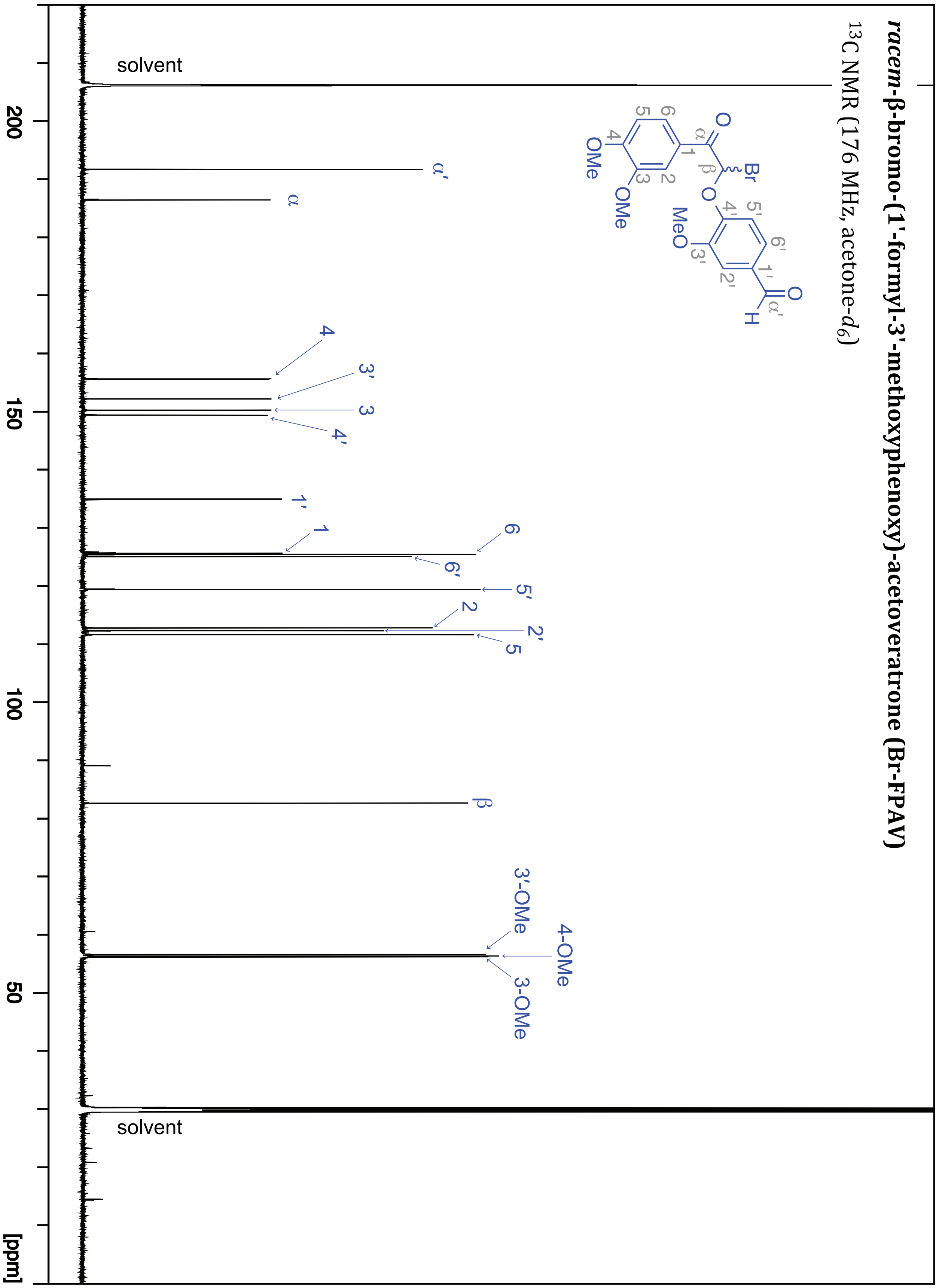

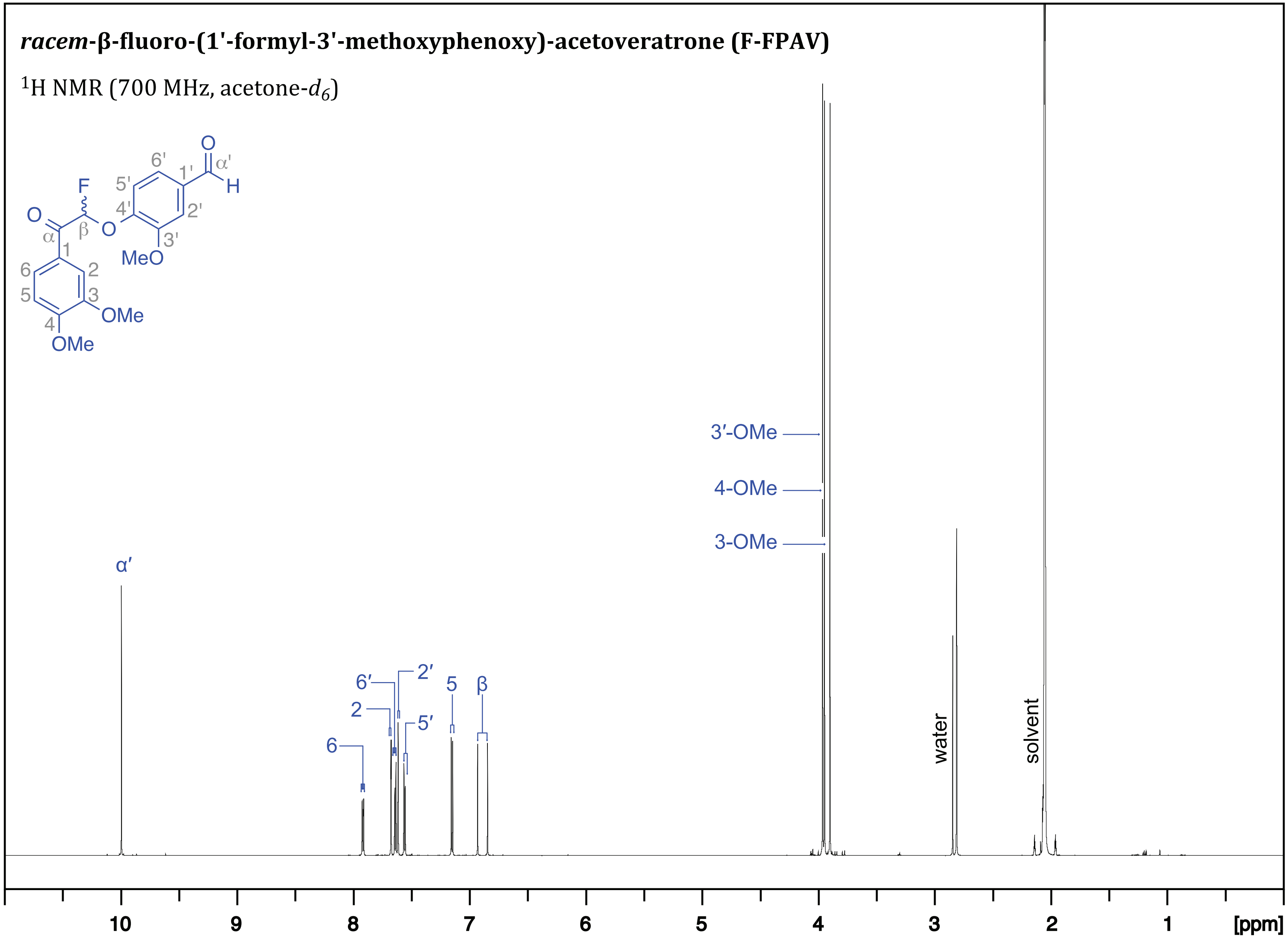
racem-β-fluoro-(1'-formyl-3'-methoxyphenoxy)-acetoveratrone (F-FPAV)
1H NMR (700 MHz, acetone-d6)
α′
6
2
6′
2′
5′
5
β
3′-OMe
4-OMe
3-OMe
water
solvent
10
9
8
7
6
5
4
3
2
1
[ppm]

racem-β-fluoro-(1'-formyl-3'-methoxyphenoxy)-acetoveratrone (F-FPAV)

^{13}C NMR (176 MHz, acetone-d_6)

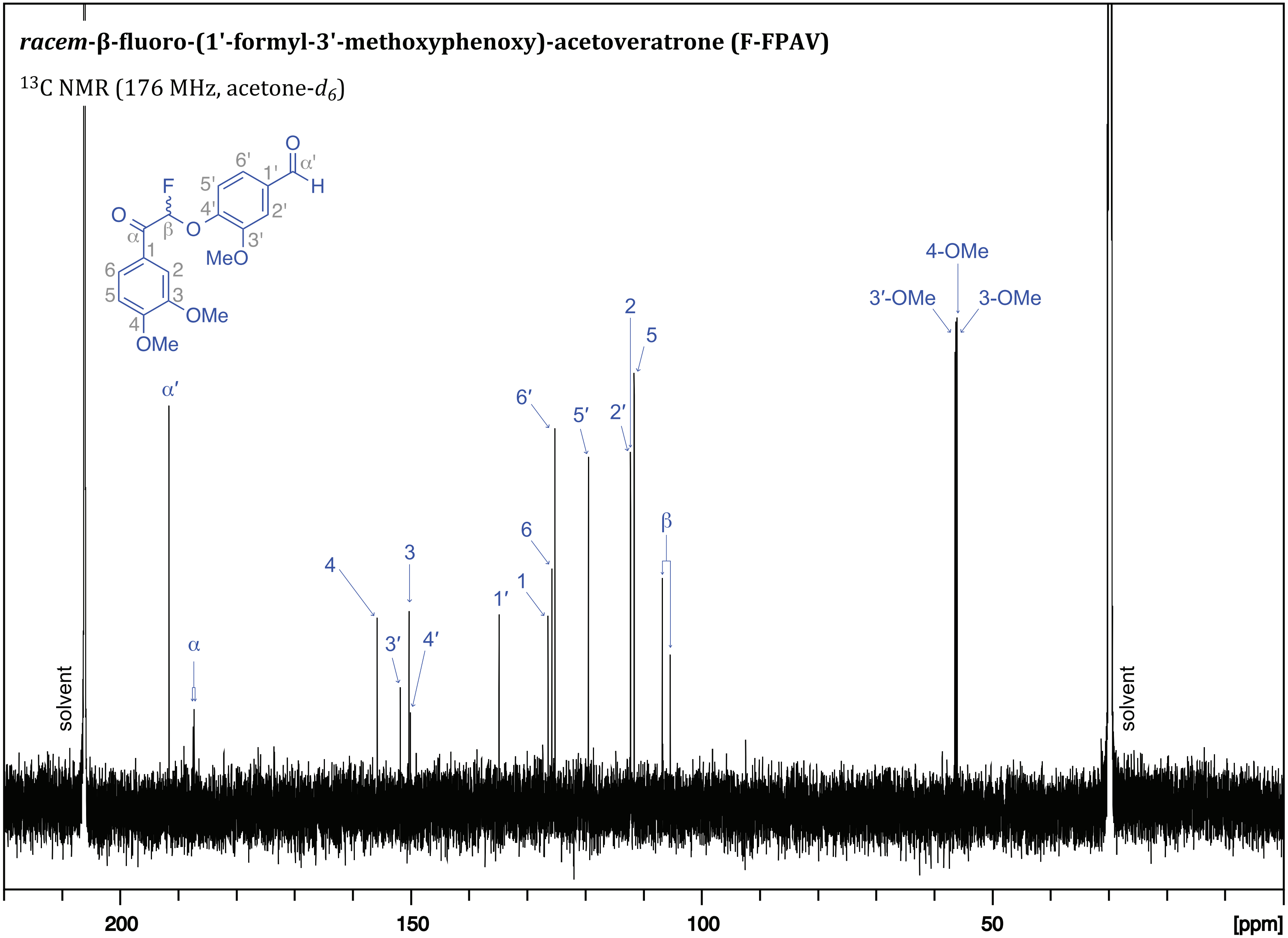

***racem*-β-fluoro-(1'-formyl-3'-methoxyphenoxy)-acetoveratrone (F-FPAV)**

^{19}F NMR (376 MHz, acetone-d_6)

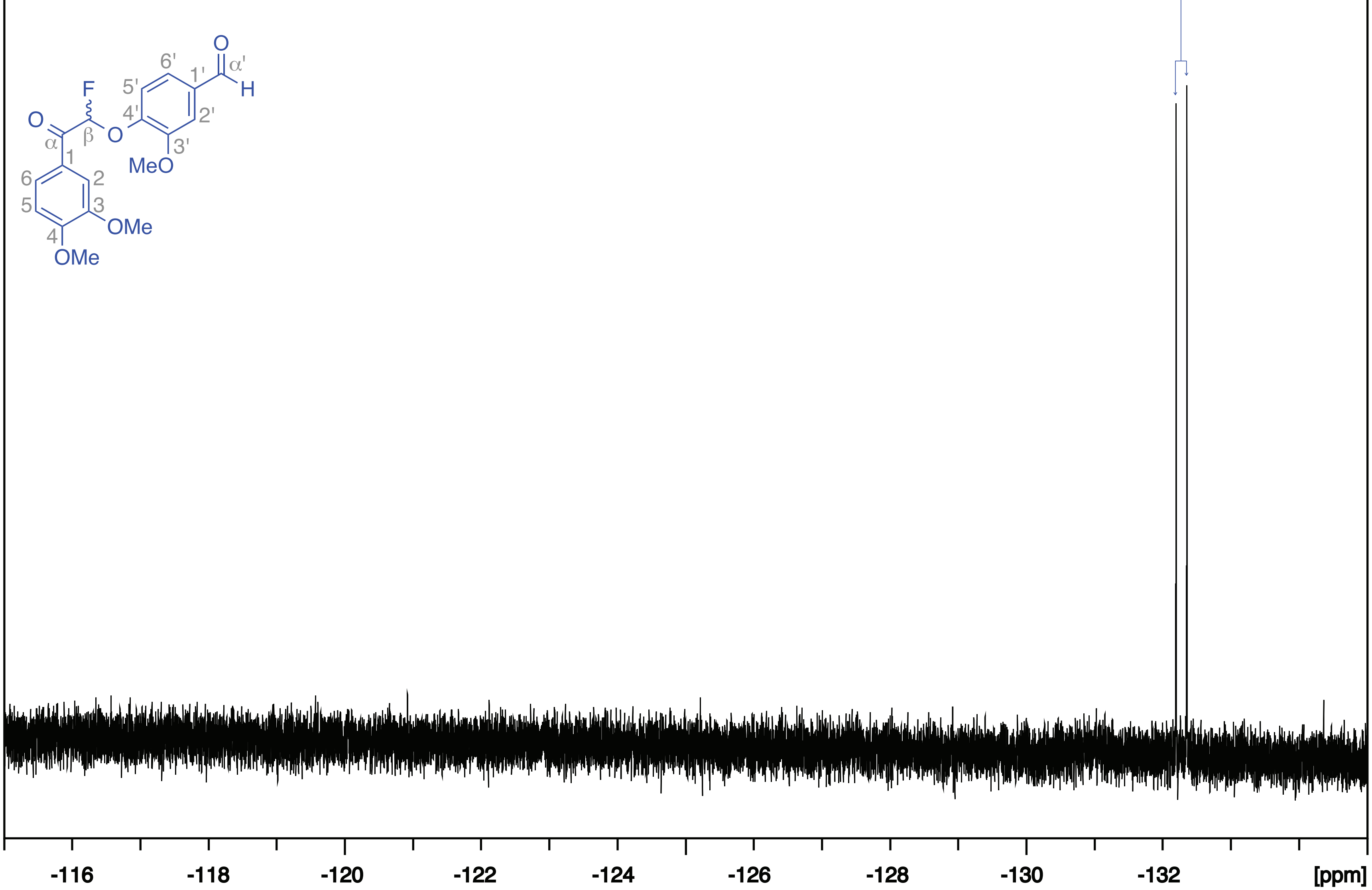

racem-β-fluoro-(1'-formyl-3'-methoxyphenoxy)-γ-hydroxypropioveratrone (F-FPHPV)

^{1}H NMR (700 MHz, acetone-d_6)

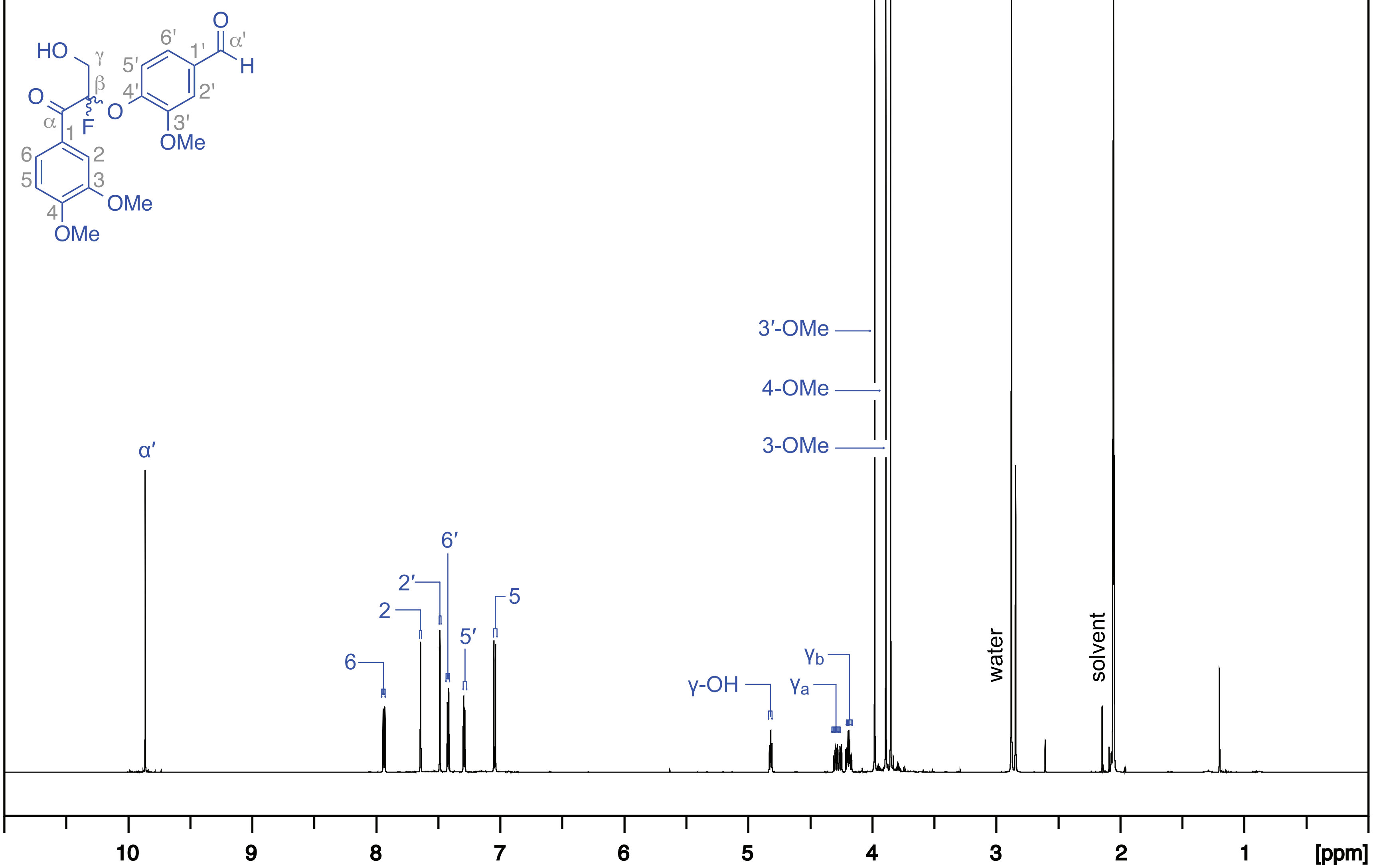

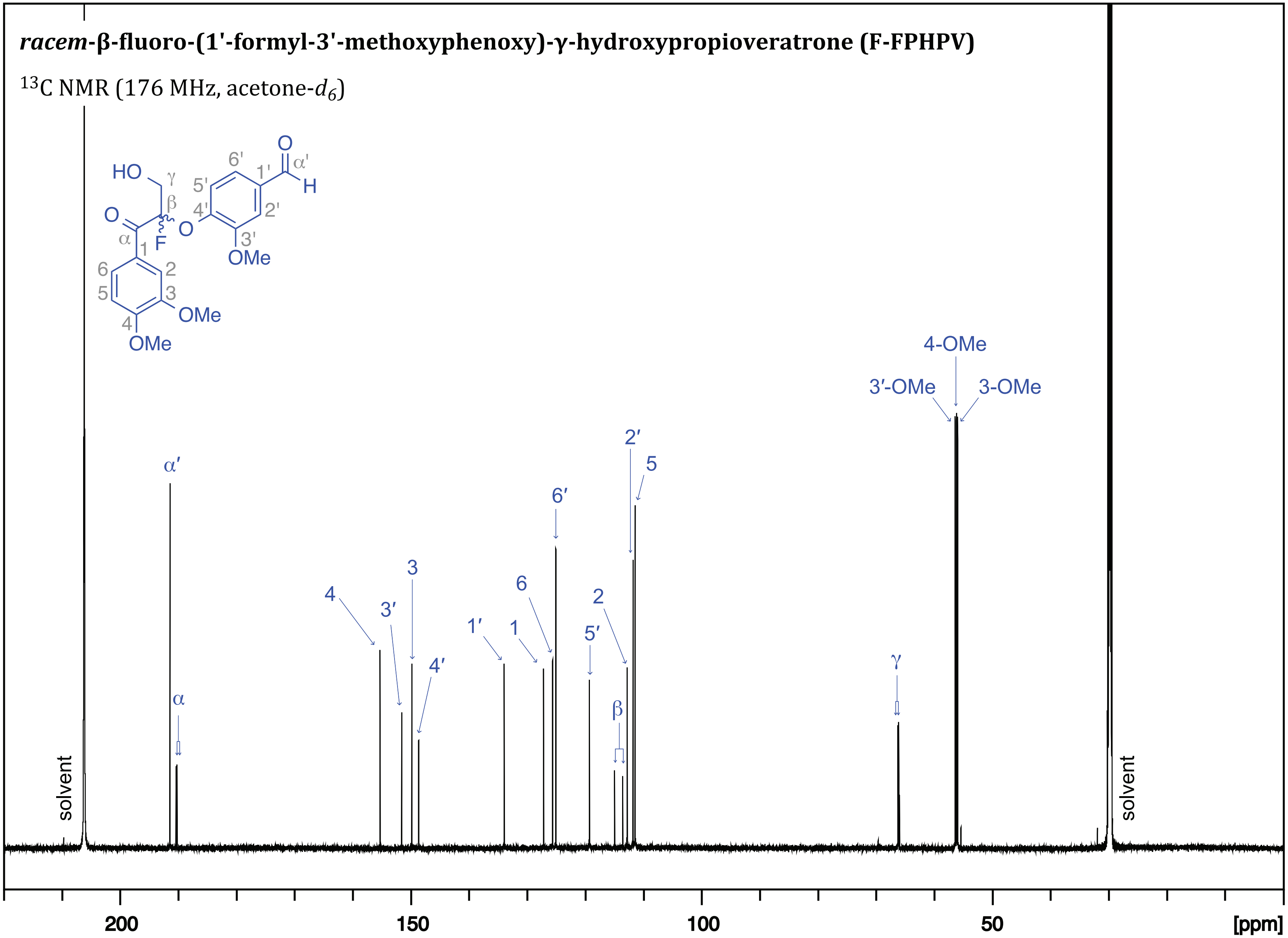
racem-β-fluoro-(1'-formyl-3'-methoxyphenoxy)-γ-hydroxypropioveratrone (F-FPHPV)
^{13}C NMR (176 MHz, acetone-d_6)
HO
γ
β
O
α
F
1
2
3
4
5
6
OMe
OMe
O
4'
5'
6'
1'
2'
3'
OMe
O
α'
H
solvent
α'
α
4
3'
3
4'
1'
1
6
6'
5'
2
β
2'
5
γ
4-OMe
3'-OMe
3-OMe
solvent
200
150
100
50
[ppm]

***racem*-β-fluoro-(1'-formyl-3'-methoxyphenoxy)-γ-hydroxypropioveratrone (F-FPHPV)**

^{19}F NMR (376 MHz, acetone-d_6)

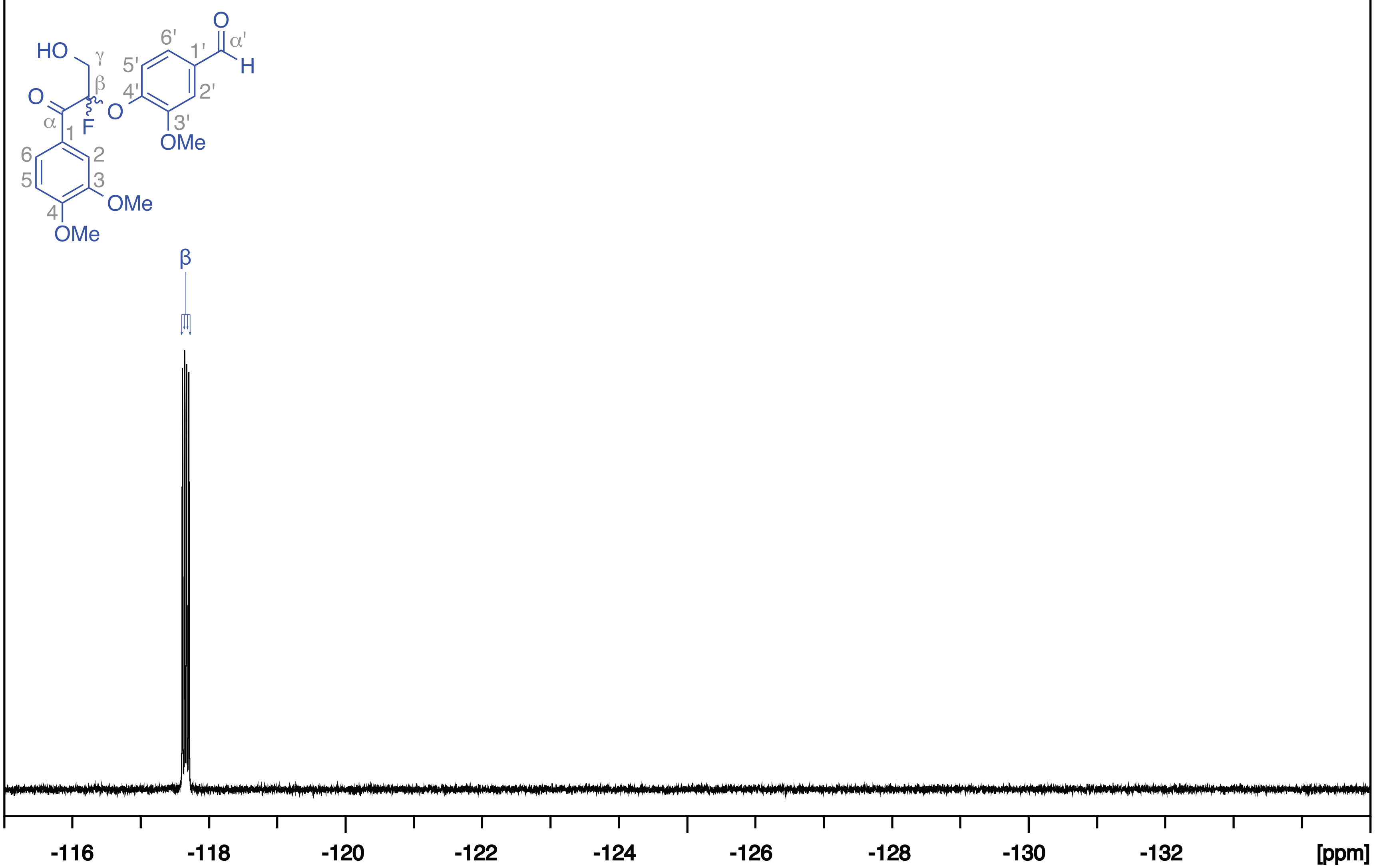

β(*S*)-fluoro-glutathionyl-γ-hydroxy-α-veratrylpropanone (β(*S*)-F-GS-HVP), from LigE

^{1}H NMR (700 MHz, D_2O)

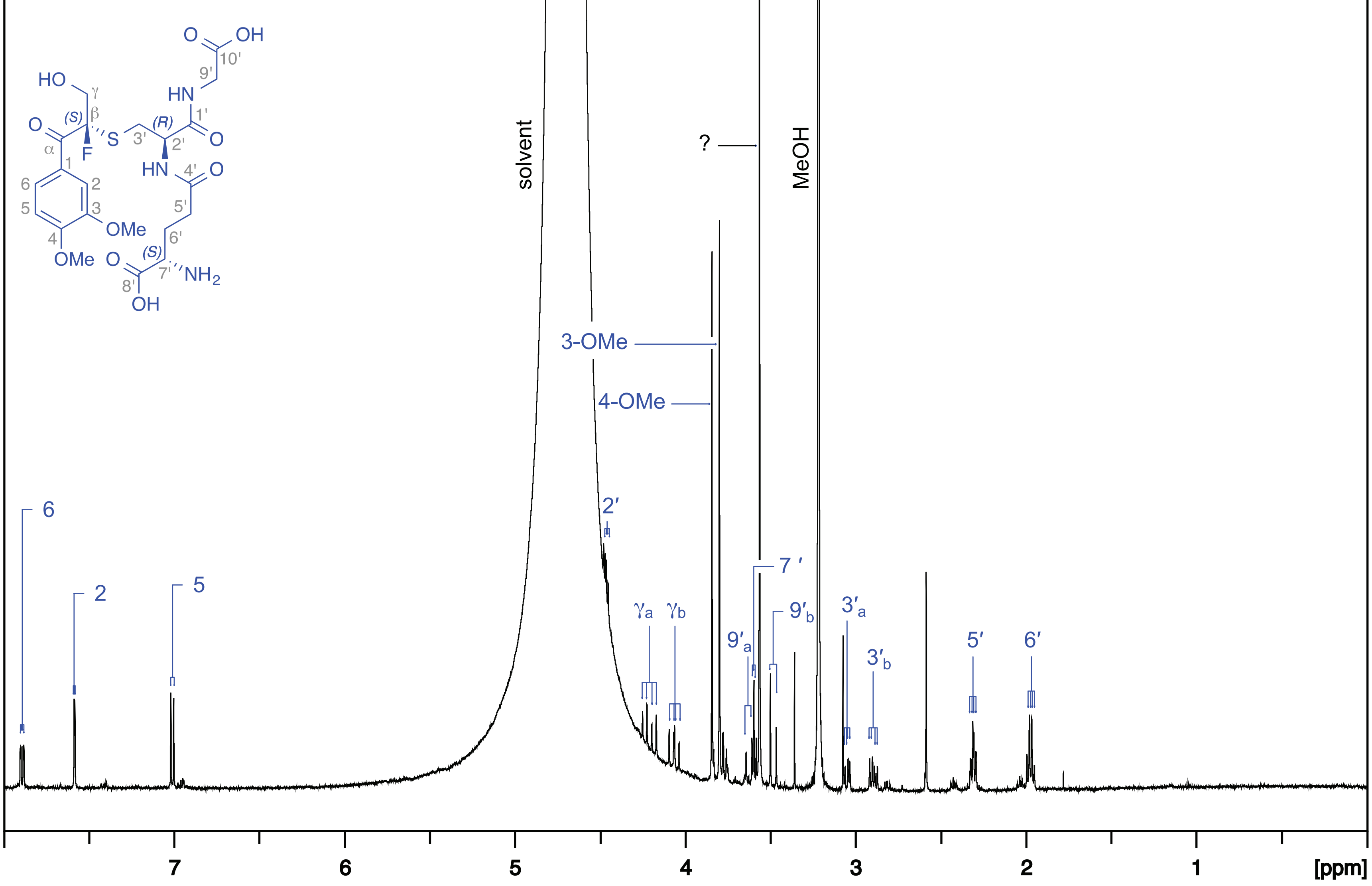

β(*S*)-glutathionyl-γ-hydroxy-α-veratrylpropanone (β(*S*)-GS-HVP), from LigE

^{1}H NMR (700 MHz, D_2O)

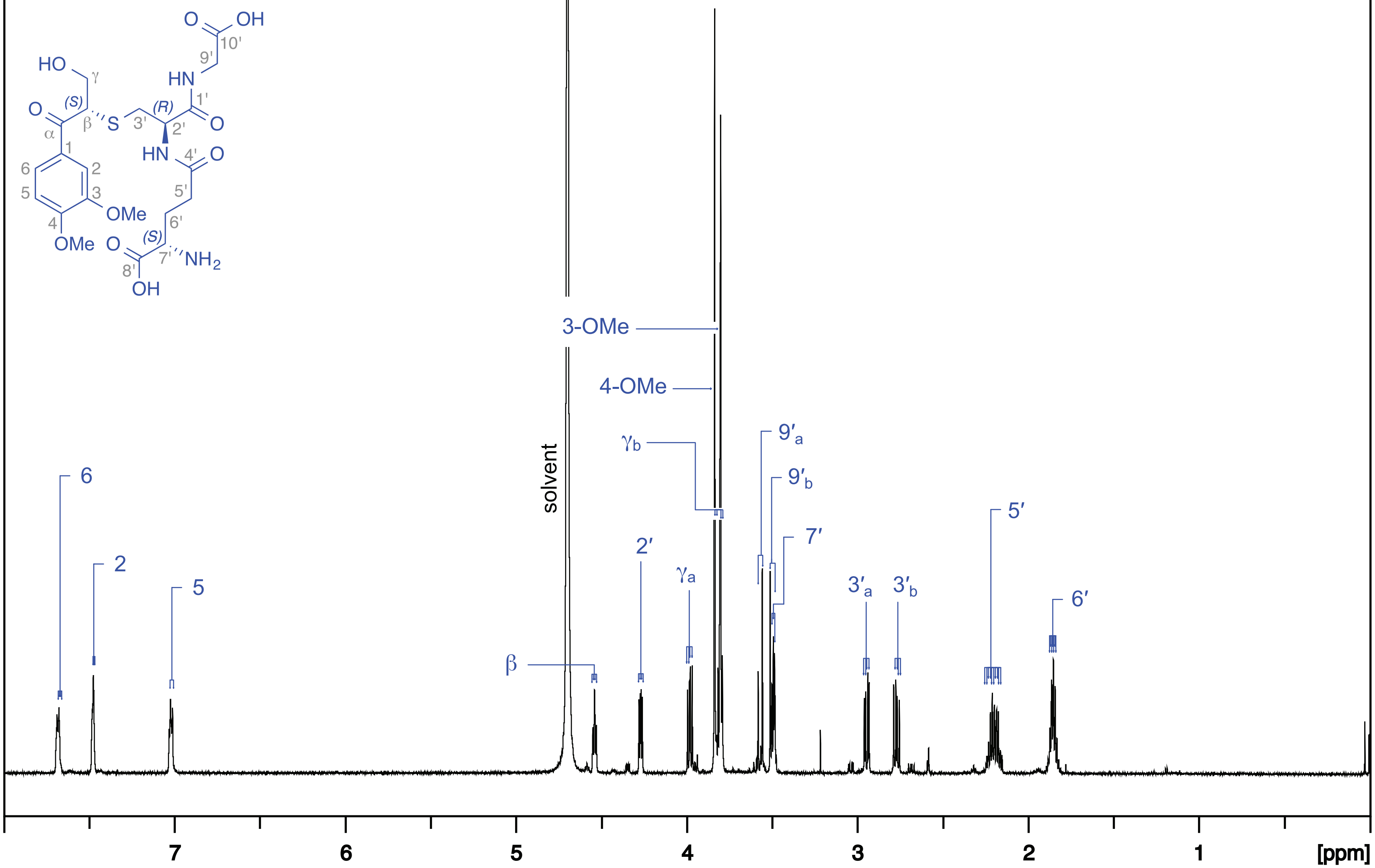

β(*S*)-glutathionyl-γ-hydroxy-α-veratrylpropanone (β(*S*)-GS-HVP), from LigE

^{13}C NMR (176 MHz, D_2O)

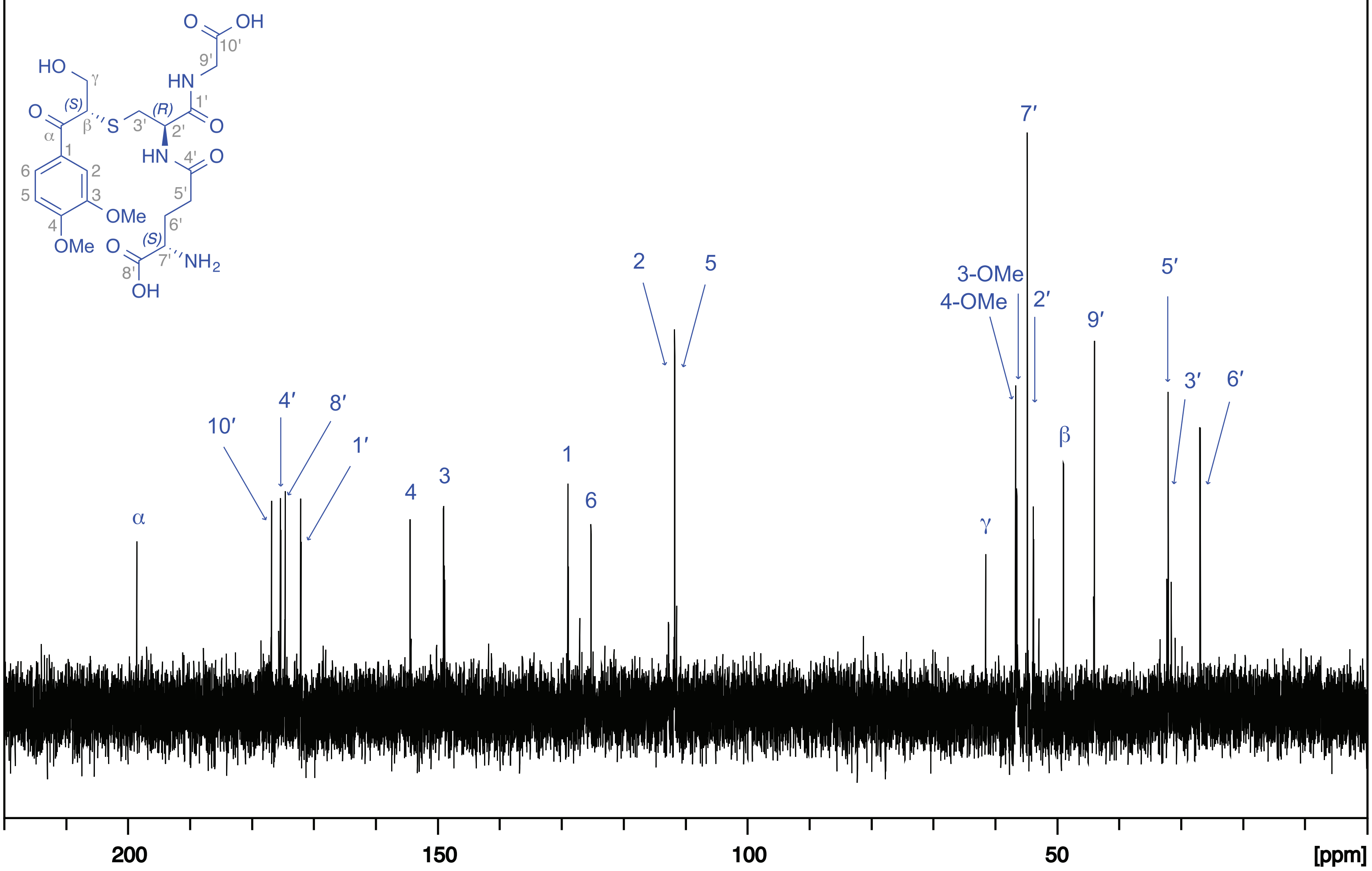

Supplement: Supplemental Data [file 10.1074_M115.694307_jbc.M115.694307-1.pdf]
